# Transdifferentiation is temporally uncoupled from progenitor pool expansion during hair cell regeneration in the zebrafish inner ear

**DOI:** 10.1242/dev.202944

**Published:** 2024-08-13

**Authors:** Marielle O. Beaulieu, Eric D. Thomas, David W. Raible

**Affiliations:** ^1^ Molecular and Cellular Biology Graduate Program, University of Washington, Seattle, WA 98195, USA; ^2^ Virginia Merrill Bloedel Hearing Research Center, Department of Otolaryngology - Head and Neck Surgery, University of Washington, Seattle, WA 98195, USA; ^3^ Neuroscience Graduate Program, University of Washington, Seattle, WA 98195, USA; ^4^ Department of Biological Structure, University of Washington, Seattle, WA 98195, USA

**Keywords:** Zebrafish, Inner ear, Hair cell, Regeneration, Transdifferentiation

## Abstract

Death of mechanosensory hair cells in the inner ear is a common cause of auditory and vestibular impairment in mammals, which have a limited ability to regrow these cells after damage. In contrast, non-mammalian vertebrates, including zebrafish, can robustly regenerate hair cells after severe organ damage. The zebrafish inner ear provides an understudied model system for understanding hair cell regeneration in organs that are highly conserved with their mammalian counterparts. Here, we quantitatively examine hair cell addition during growth and regeneration of the larval zebrafish inner ear. We used a genetically encoded ablation method to induce hair cell death and we observed gradual regeneration with correct spatial patterning over a 2-week period following ablation. Supporting cells, which surround and are a source of new hair cells, divide in response to hair cell ablation, expanding the possible progenitor pool. In parallel, nascent hair cells arise from direct transdifferentiation of progenitor pool cells temporally uncoupled from supporting cell division. These findings reveal a previously unrecognized mechanism of hair cell regeneration with implications for how hair cells may be encouraged to regenerate in the mammalian ear.

## Teaser

**Summary:** Hair cell regeneration in the zebrafish inner ear occurs through transdifferentiation after a transient wave of supporting cell proliferation that expands the precursor pool.

## INTRODUCTION

The sensory organs of the inner ear that detect sound and head position are highly conserved across the vertebrate kingdom. The potential to regenerate these organs, however, is not as widespread. Hair cells, the mechanosensory cells of the inner ear, are particularly fragile and are vulnerable to death caused by exposure to ototoxic drugs, injury and age-related degeneration. Although mammals can regenerate hair cells at perinatal stages (Burns et al., 2012a; Mellado Lagarde et al., 2014; White et al., 2006), this ability declines rapidly after birth (Burns et al., 2012b; Cox et al., 2014; Maass et al., 2015). By adulthood, regeneration is limited in mammalian vestibular organs (Bucks et al., 2017; Forge et al., 1993; Golub et al., 2012; Kawamoto et al., 2009) and completely lost in the auditory system (Oesterle et al., 2008). As a result, hair cell death can lead to permanent auditory and vestibular deficits in humans. In contrast, many other vertebrates, including fish, amphibians and birds, can regenerate functional hair cells throughout life (Avallone et al., 2008; Baird et al., 1996; Corwin and Cotanche, 1988; Cruz et al., 1987, 2015; Harris et al., 2003; Jimenez et al., 2021; Jones and Corwin, 1996; Lombarte et al., 1993; Ryals and Rubel, 1988; Smith et al., 2006; Taylor and Forge, 2005; Weisleder and Rubel, 1992).

Zebrafish are well known for their regenerative potential and are commonly used to study hair cell development, death and regeneration (reviewed by Pickett and Raible, 2019; Sheets et al., 2021). In addition to inner ear hair cells, fish and amphibians have analogous hair cells in an external sensory system called the lateral line, which is used to detect changes in water flow for behaviors such as schooling and predator evasion. Much of our current understanding of zebrafish hair cell function and regeneration comes from studies of the lateral line, whereas zebrafish inner ear hair cells have been relatively understudied. The zebrafish inner ear remains a promising model system for studying hair cell regeneration due to its high level of conservation with the inner ear of mammals, and to the extensive genetic and imaging tools available for zebrafish.

**Fig. 1.**
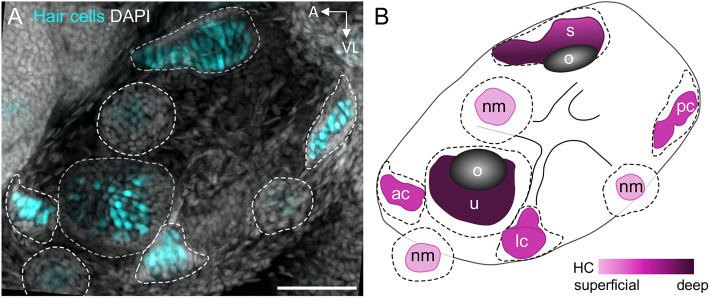
**Inner ear organs of the larval zebrafish.** (A) Maximum intensity projection image of *Tg(myo6b:GFP)* 5 dpf larval zebrafish ear. GFP-labeled hair cells are shown in cyan and DAPI-labeled nuclei are shown in gray. Dotted outlines delineate neuromast and inner ear organ boundaries. Scale bar: 50 µm. (B) Diagram of a 5 dpf larval zebrafish ear. Color gradient indicates depth of organs, where lighter colors indicate more superficial structures and darker colors indicate deeper structures. Dotted outlines delineate neuromast and inner ear organ boundaries, while color-filled areas indicate location of hair cells. ac, anterior crista; lc, lateral crista; nm, neuromast; o, otolith; pc, posterior crista; s, saccule; u, utricle.

Zebrafish share several conserved inner ear organs with other vertebrates: three cristae, which sense angular rotation of the head within the semicircular canals; and two otolith organs, or maculae: the utricle and saccule (Fig. 1). In mammals, the utricle and saccule sense gravity and linear acceleration, while an additional structure, the cochlea, is highly specialized for hearing. Zebrafish do not have a cochlea; instead, auditory function is distributed across the macular organs, with the saccule likely playing an outsized role (Breitzler et al., 2020; Schuck and Smith, 2009). Only the utricle is indispensable for vestibular function (Riley and Moorman, 2000), but both macular organs have some capacity to respond to both auditory and vestibular stimuli (Favre-Bulle et al., 2020; Popper and Fay, 1993; Yao et al., 2016).

Within specific vestibular sensory organs, hair cells can be divided into zones based on differences in morphology, physiology, innervation and gene expression (Burns and Stone, 2017; Eatock and Songer, 2011). The maculae can be divided into a central striolar region and more peripheral extrastriolar regions, with phasic striolar hair cells sensitive to higher-frequency input compared with tonic extrastriolar cells. Fishes, including zebrafish, also have striolar and extrastriolar hair cells analogous to those of other vertebrates, as defined by shared morphological characteristics (Chang et al., 1992; Jiang et al., 2017; Liu et al., 2022; Platt, 1993), physiological responses (Tanimoto et al., 2022) and gene expression (Shi et al., 2023). The cristae are also organized into central and peripheral zones, with molecularly and morphologically distinct hair cells (Bang et al., 2001; Haddon and Lewis, 1996; Shi et al., 2023; Smith et al., 2020; Zhu et al., 2021).

Hair cells are surrounded by and interspersed with supporting cells that perform many crucial roles during the life and death of hair cells (Wan et al., 2013), including acting as a source of new hair cells (Corwin and Cotanche, 1988; Lin et al., 2011; Lopez-Schier and Hudspeth, 2006; Millimaki et al., 2010; Weisleder et al., 1995). The mechanism by which hair cells are regenerated differs by model system, with a crucial point of difference being whether precursors divide before giving rise to new hair cells. In the lateral line, nascent hair cells are added in pairs as a result of symmetric division and differentiation of supporting cells (Lopez-Schier and Hudspeth, 2006; Mackenzie and Raible, 2012; Romero-Carvajal et al., 2015; Wibowo et al., 2011). When regeneration is observed in mature mammalian vestibular organs, hair cells are added by direct transdifferentiation of supporting cells (Golub et al., 2012). A dual mechanism has been observed in the auditory organ of birds, whereby hair cells are regenerated in an initial wave of transdifferentiation followed by a later wave of asymmetric proliferation (Roberson et al., 1996, 2004). Previous studies have demonstrated hair cell regeneration in the zebrafish inner ear (Jimenez et al., 2021; Millimaki et al., 2010; Schuck and Smith, 2009), with evidence for both proliferative replacement and transdifferentiation; however, definitive experiments are lacking. The transdifferentiation hypothesis is supported by recent single cell and nucleus RNA-seq data, which suggest that the inner ear does not have a clear mitotically cycling supporting cell population, as is seen in the lateral line (Baek et al., 2022; Lush et al., 2019), and instead show a substantial transition state population during regeneration that shares gene expression aspects of both hair cells and supporting cells (Jimenez et al., 2022).

Here, we describe a mechanism of hair cell regeneration in the zebrafish inner ear in which supporting cell proliferation in response to hair cell death is not directly coupled with the differentiation of regenerating hair cells. First, we used transgenic zebrafish lines to determine the timecourse of hair cell addition during larval zebrafish development. We found that hair cells are added throughout the larval stage of development, and that few hair cells are removed due to hair cell turnover during this time. Both hair cell subtypes of the cristae are added at equivalent rates, with some cells converting from peripheral to central subtype over time, resulting in maintenance of organ patterning. When crista hair cells were ablated, hair cell numbers recovered relatively slowly over the course of 2 weeks, central-type hair cells were produced at an increased rate and proper organ patterning ultimately recovered. We provide evidence that most regenerating hair cells are formed by transdifferentiation. We find that ablation causes an initial burst of supporting cell division, but new hair cells are not differentially derived from this dividing population. Rather, hair cell numbers recover during regeneration due to a transient increase in supporting cell number, contributing to an expanding progenitor pool size.

## RESULTS

### Zebrafish inner ear sensory patches grow constantly during the larval stage

Sensory patches in the fish inner ear add new hair cells continuously throughout the life of an animal (Bang et al., 2001; Corwin, 1981, 1983; Higgs et al., 2002, 2003). To distinguish hair cell regeneration from addition during growth, we first quantified hair cell addition under homeostatic conditions. We examined the larval stage, during which the inner ear organs become functional and remain superficial enough for imaging in intact fish. Variations in environmental factors greatly affect fish growth. After 5 days post-fertilization (dpf), standard length (SL), a measurement from the snout tip to the caudal peduncle, becomes a better indicator of developmental stage than time (Parichy et al., 2009). The larval stage begins at 72 h post-fertilization and continues until 30-45 dpf, when the SL of the fish is 11 mm. The utricle is formed and functional by 4 dpf (Mo et al., 2010; Riley and Moorman, 2000), and contains both striolar and extrastriolar type hair cells. The cristae do not become functional until later on, when the larvae are 8 mm in SL, around 30 dpf (Beck et al., 2004), when the semicircular canals are large enough to allow adequate fluid flow to stimulate hair cells. The cristae, however, are formed by 5 dpf, and contain both central and peripheral hair cell subtypes (Bang et al., 2001; Haddon and Lewis, 1996; Shi et al., 2023; Zhu et al., 2021).

**Fig. 2.**
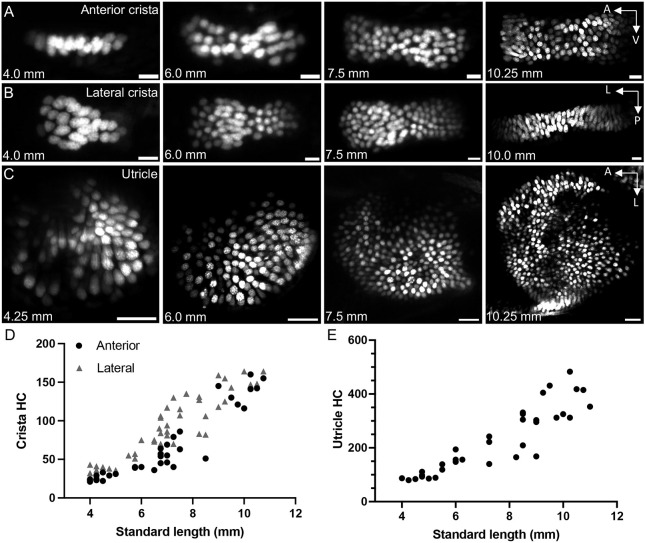
**Addition of hair cells during larval zebrafish growth.** (A) Maximum intensity projections of *Tg(myo6b:NLS-Eos)* anterior crista hair cells at standard lengths 4.0 mm, 6.0 mm, 7.5 mm and 10.25 mm. Scale bars: 10 μm. (B) Maximum intensity projections of lateral crista hair cells at standard lengths 4.0 mm, 6.0 mm, 7.5 mm and 10.0 mm. Scale bars: 10 μm. (C) Maximum intensity projections of utricle hair cells at standard lengths 4.25 mm, 6.0 mm, 7.5 mm and 10.25 mm. Scale bars: 20 μm. (D) Quantification of hair cell number in the anterior and lateral cristae across the larval stage of development. Anterior crista data points are represented by black circles (*n*=35), whereas the lateral crista results are represented by gray triangles (*n*=47). Each data point represents one ear from one fish. (E) Quantification of utricle hair cell number across the larval stage (*n*=34).

To determine baseline hair cell addition in the zebrafish inner ear, we used a *Tg(myo6b:nls-Eos)* (Cruz et al., 2015) transgenic zebrafish, which expresses the photoconvertible protein Eos in hair cell nuclei. In both cristae and utricle, hair cells were added steadily across the larval stage (Fig. 2). Among the cristae, the lateral crista is the earliest to form and is slightly larger than the anterior and posterior cristae at the beginning of the larval stage. This size discrepancy continues over time, while the anterior and posterior cristae remain similar in size (Fig. 2D, Fig. S1). Owing to its similarity in size to the anterior crista and depth in larger fish, the posterior crista was not a focus of subsequent experiments. These results indicate that hair cells are added consistently in each of the sensory organs as larvae grow.

### Little hair cell turnover occurs in the developing inner ear organs

**Fig. 3.**
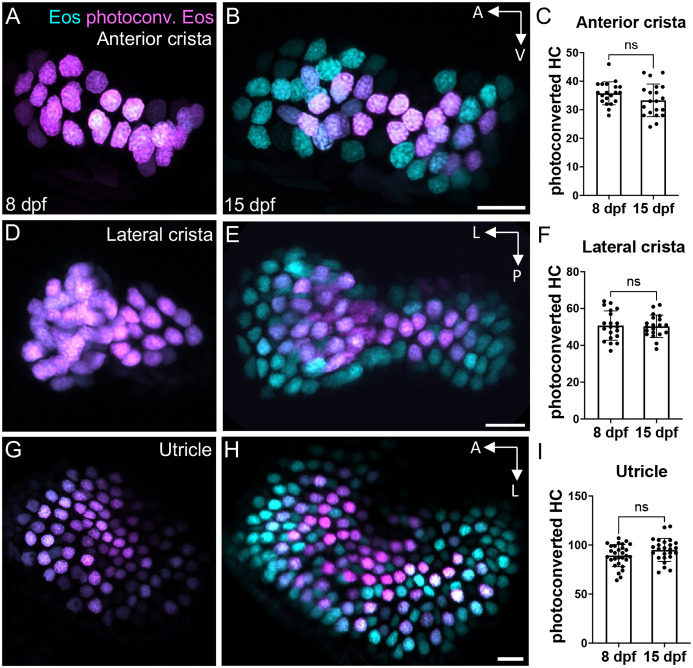
**Little hair cell turnover occurs in the larval zebrafish ear.** (A,B) Representative maximum intensity projection images of *Tg(myo6b:NLS-Eos)* anterior cristae (A) immediately post-photoconversion at 8 days post-fertilization (dpf) or (B) 1 week post-photoconversion at 15 dpf. Hair cells that were photoconverted retained photoconverted (magenta) Eos signal, while new hair cells have unconverted (cyan) Eos only. (C) Quantification of anterior crista photoconverted hair cells at 8 and 15 dpf (*n*=20 at 8 dpf, 20 at 15 dpf). (D-I) Analogous results for the lateral crista (*n*=20, 20) (D-F) and for the utricle (*n*=29, 25) (G-I). Unpaired *t*-tests indicate no significant difference between the number of photoconverted hair cells at these two timepoints (anterior crista *P*=0.125, lateral crista *P*=0.859, utricle *P*=0.071). Scale bars: 10 µm. Data are mean±s.d.

Hair cells regularly turn over in the adult zebrafish lateral line, with a half-life of approximately 1 week (Cruz et al., 2015). Studies from birds and mice suggest that the rate of turnover varies across species (Bucks et al., 2017; Goodyear et al., 1999; Jørgensen and Mathiesen, 1988; Kil et al., 1997). To determine the rate of turnover in the zebrafish inner ear, we again used the *Tg(myo6b:nls-Eos)* line. Eos exhibits an irreversible green to red photoconversion upon exposure to UV light. Larval fish were placed under UV light for 10 min at 8 dpf (SL 4.0-4.5) and fixed and imaged either immediately after photoconversion (Fig. 3A,D,G) or after 1 week of growth (Fig. 3B,E,H). Hair cells that are added post-photoconversion can be identified by the absence of photoconverted Eos in their nuclei, whereas older cells retain the converted Eos signal. The anterior crista, lateral crista and utricle showed no significant decrease in photoconverted hair cell nuclei over the course of 1 week (Fig. 3C,F,I). This experiment was repeated for the subsequent week of growth, from 14 to 21 dpf, again with no discernable decrease in photoconverted hair cell number (Fig. S2). Together, these results indicate that little to no hair cell turnover occurs in the zebrafish inner ear organs during the early larval stage.

### Two hair cell subtypes are added consistently during growth

**Fig. 4.**
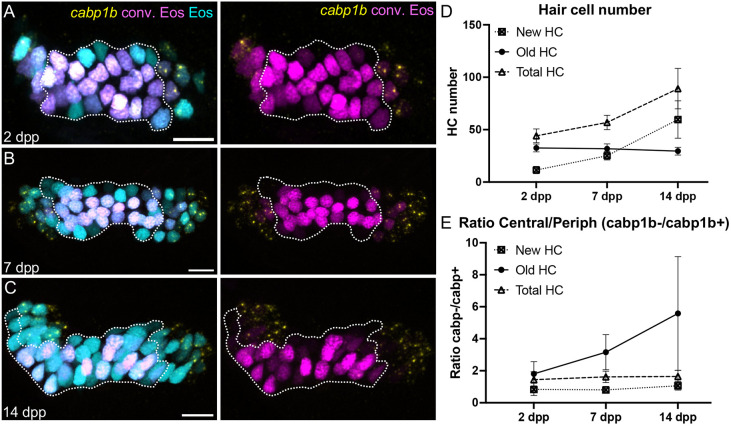
**Identification of inner ear hair cell subtypes during larval growth.** (A-C) Maximum intensity projection images of HCR-FISH probing for *cabp1b* expression in *Tg(myo6b:NLS-Eos)* anterior cristae at (A) 2 days post-photoconversion (dpp) (10 dpf, *n*=14); (B) 7 days dpp (15 dpf, *n*=12); and (C) 14 dpp (22 dpf, *n*=8). Old hair cells retain photoconverted (magenta) Eos signal while new hair cells have unconverted (cyan) Eos only. Peripheral-type hair cells are labeled by the *cabp1b* HCR probe (yellow). Dotted outline delineates the central *cabp1b*^−^ region of the sensory patch. Scale bars: 10 µm. (D) Increase in hair cell numbers over the course of the experiment. (E) Ratio of central (*cabp1^−^*) to peripheral (*cabp1b*^+^) hair cells over time. The increased ratio for old cells suggests phenotypic conversion from peripheral to central hair cell type over time. Data are mean±s.d.

We wanted to understand how the makeup of sensory organs changes as new hair cells are added. By the larval stage, central and peripheral subtypes exist in the cristae, and striolar and extrastriolar cells are present in the maculae (Qian et al., 2022; Shi et al., 2023; Smith et al., 2020, 2023; Tanimoto et al., 2022). We have previously identified marker genes for hair cell subtypes that can be used in hybridization chain reaction fluorescence *in situ* hybridization (HCR-FISH) (Choi et al., 2016; Shi et al., 2023). Here, we used probes against *cabp1b* to label peripheral cells in the cristae. We photoconverted *Tg(myo6b:nls-Eos)* fish at 8 dpf and fixed fish at three subsequent timepoints for imaging: 2 days post-photoconversion (dpp), 7 dpp and 14 dpp. HCR-FISH was then performed with *cabp1b* probes to distinguish subtypes of ‘new’ (cyan) from ‘old’ (magenta+cyan) hair cells (Fig. 4A-C). During this period, there is a substantial increase in the number of new hair cells with little change in old hair cells (Fig. 4D). Based on the spatial pattern of hair cell addition occurring around the perimeter, we hypothesized that peripheral subtype hair cells would make up the majority of new hair cells. In fact, although *cabp1b^+^* new hair cells were common at the peripheral poles of the crista, an almost equal percentage of new central-type *cabp1b^−^* hair cells were added. This even split of new central and peripheral hair cells was consistent at each timepoint examined (Fig. 4A-C,E), indicating that both subtypes are added at relatively constant rates. When examining the identity of old hair cells, we observed an increase in the fraction of central to peripheral-type cells over time (Fig. 4A-C,E). The gene *scn5lab* is expressed in the inverse pattern of *cabp1b*, and preferentially labels centrally located crista hair cells (Fig. S3A). When HCR-FISH was performed with probes for *scn5lab*, the vast majority of photoconverted hair cell nuclei are located within the central, scn5lab^+^ region by 14 dpp (Fig. S3B). Together these results suggest that some hair cells convert from peripheral identity to central identity as sensory patches grow larger, resulting in a consistent overall ratio of central to peripheral cells.

### Crista hair cells are regenerated in the week following ablation

**Fig. 5.**
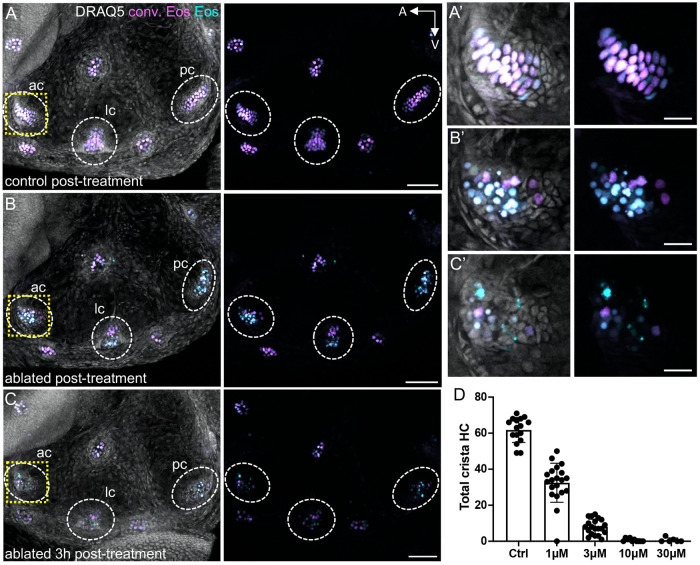
**Trpv1-capsaicin hair cell ablation.** (A) Maximum intensity projection of a photoconverted 8 dpf *Tg(myo6b:NLS-Eos)* larval inner ear immediately after 1 h of treatment with 10 μM capsaicin. (B,C) Maximum intensity projection of a sibling *Tg(myo6b:NLS-Eos);Tg(myo6b:TrpV1-mClover)* inner ear immediately after 1 h of capsaicin treatment (B) or 3 h after capsaicin wash out (C). Images show photoconverted (magenta) and unconverted (cyan) Eos signal with and without DRAQ5-labeled nuclei. Dashed oval regions indicate anterior, lateral and posterior cristae. The areas outlined by a dashed yellow line indicate the magnified anterior cristae regions shown in A′-C′. (D) Dose-response curve for hair cells at 5 dpf after 1 h of treatment with capsaicin at different concentrations. Control treatment represents DMSO alone. Each data point represents the number of hair cells in combined anterior, lateral and posterior crista of one fish ear (*n*=6-20). Data are mean±s.d. Scale bars: 50 µm (A-C); 10 µm (A′-C′).

Unlike in the lateral line, hair cells in the inner ear are protected from ototoxic drugs administered through the water, which are unable to diffuse into the ear. To overcome this limitation, we designed a *Tg(myo6b:TrpV1-mClover)* transgenic line where the mammalian TRPV1 channel is expressed in target cells (Chen et al., 2016). When exposed to its ligand capsaicin, the mammalian TRPV1 channel opens, resulting in cell death by cation influx. Endogenous zebrafish Trpv1 is unresponsive to capsaicin, like other non-mammalian forms (Gau et al., 2013). Expressing mammalian TRPV1 under a hair cell-specific promoter and exposing the fish to capsaicin results in quick and effective hair cell death in the cristae. Expression of TRPV1 in the absence of capsaicin does not significantly alter the number of hair cells in the cristae (Fig. S4A). We crossed the *Tg(myo6b:TrpV1-mClover)* line to the *Tg(myo6b:nls-Eos)* to better visualize hair cell nuclei. Larvae were treated with 10 μM capsaicin for 1 h at 8 dpf, immediately after which hair cell debris was observed across all three cristae (Fig. 5A-B). Few crista hair cells survived the capsaicin treatment in ablated condition fish (Fig. S4B), and supporting cell numbers were not largely impacted by hair cell loss (Fig. S4C). By 3 h post-treatment, hair cell debris had been largely cleared (Fig. 5C). Although this method is highly efficient at killing crista hair cells, hair cell death was inconsistent in the lateral line and was undetectable in the macular organs, likely as a result of different expression levels due to the genetic landscape associated with the location of transgene insertion. Therefore, we focused our subsequent regeneration experiments on the cristae. Dose response curves were performed at 5 dpf to determine the appropriate concentration of capsaicin for complete hair cell ablation (Fig. 5D); we found that a 10 μM exposure was sufficient. In all subsequent experiments, larvae were treated with 10 μM capsaicin in system water for 1 h at 8 dpf. Regeneration experiments were performed in sibling *Tg(myo6b:nls-Eos)* fish in a *nac/roy* background with and without *Tg(myo6b:TrpV1-mClover)*. Owing to the relative brightness of Eos, larvae could not be screened for mClover expression under a fluorescent dissecting microscope, even after photoconversion. Instead, fish were screened for dying hair cells immediately after capsaicin treatment; those with dying crista hair cells became the ablated group and those without dying hair cells formed the control group.

**Fig. 6.**
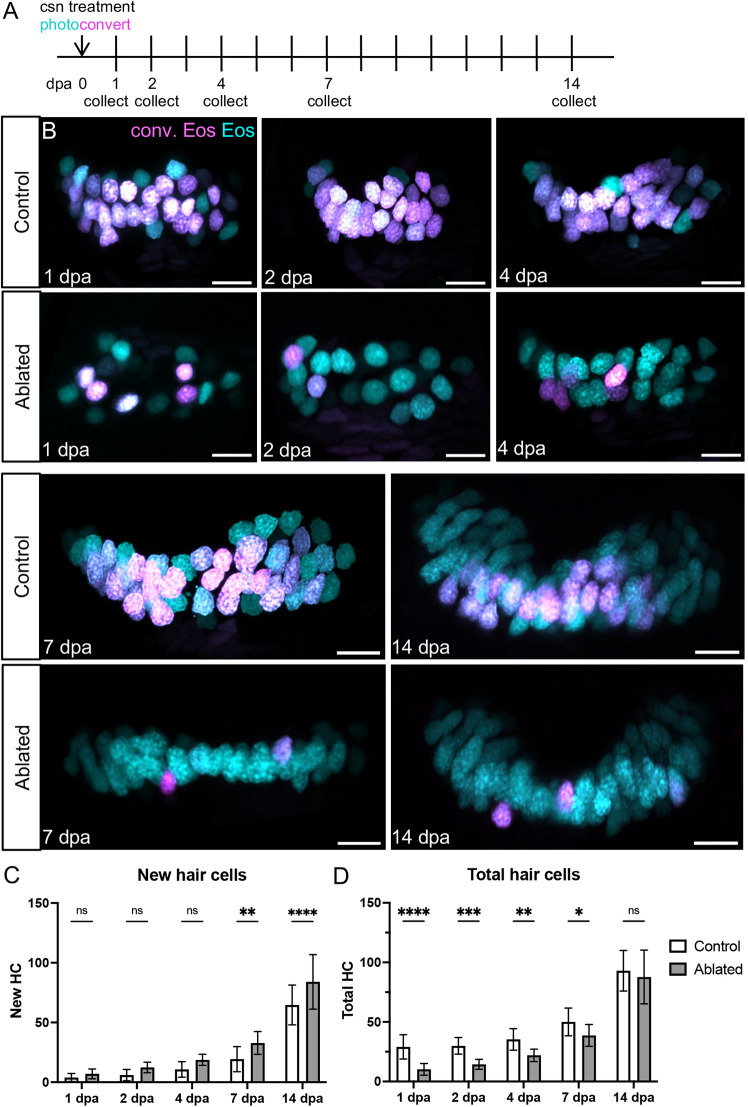
**Anterior crista hair cells regenerate during the 2 weeks that follow ablation.** (A) *Tg(myo6b:NLS-Eos)* sibling larvae with or without *Tg(myo6b:TrpV1-mClover)* were photoconverted and treated with capsaicin to ablate hair cells at 8 dpf. Larvae were collected at five timepoints over the following 2 weeks: 1 (*n*=22 control, 25 ablated), 2 (*n*=13, 20), 4 (*n*=19, 18), 7 (*n*=16, 13) or 14 (*n*=18, 15) days-post ablation. (B) Representative maximum intensity projections of anterior crista in control and ablated fish at five timepoints after treatment. Nuclei of cells that survived capsaicin treatment contain photoconverted Eos (magenta). Hair cells newly added after capsaicin treatment have nuclei with only unconverted Eos (cyan). Scale bars: 10 µm. (C) Quantification of new (cyan only) hair cells in ablated and control anterior crista. Two-way ANOVA variation across condition, *P*<0.0001; Šídák's multiple comparisons post-hoc test for 7 dpa, **adjusted *P*-value=0.0021, and for 14 dpa, ****adjusted *P*-value<0.0001. (D) Quantification of total hair cells in ablated and control anterior crista. Two-way ANOVA variation across condition, ****P<0.0001; Šídák's multiple comparisons post-hoc test for 1 dpa, ****adjusted *P*-value<0.0001, for 2 dpa, ***adjusted *P*-value=0.0006, for 4 dpa, **adjusted *P*-value=0.0015, and for 7 dpa *adjusted *P*-value=0.0342. Data are mean±s.d.

To compare hair cell addition after ablation to growth, hair cells were photoconverted and in some fish ablated at 8 dpf, then fixed at subsequent timepoints to count hair cell nuclei (Fig. 6A). In ablated anterior cristae, the number of new hair cells increased significantly compared with controls over the course of 2 weeks post-treatment (Fig. 6B,C). Correspondingly, total hair cell number was decreased after capsaicin treatment in ablated fish but slowly recovered to control levels by 14 days post-ablation (dpa) (Fig. 6D). Similar results were obtained for the lateral crista (Fig. S5). No body length difference was observed at any timepoint between control and ablated fish, suggesting that crista hair cell ablation does not affect overall growth rates (Fig. S6). The increased rate of hair cell addition and eventual recovery of hair cell numbers in ablated crista suggest that a regenerative response occurs alongside hair cell addition due to organ growth.

### Hair cell identity is maintained during regeneration

**Fig. 7.**
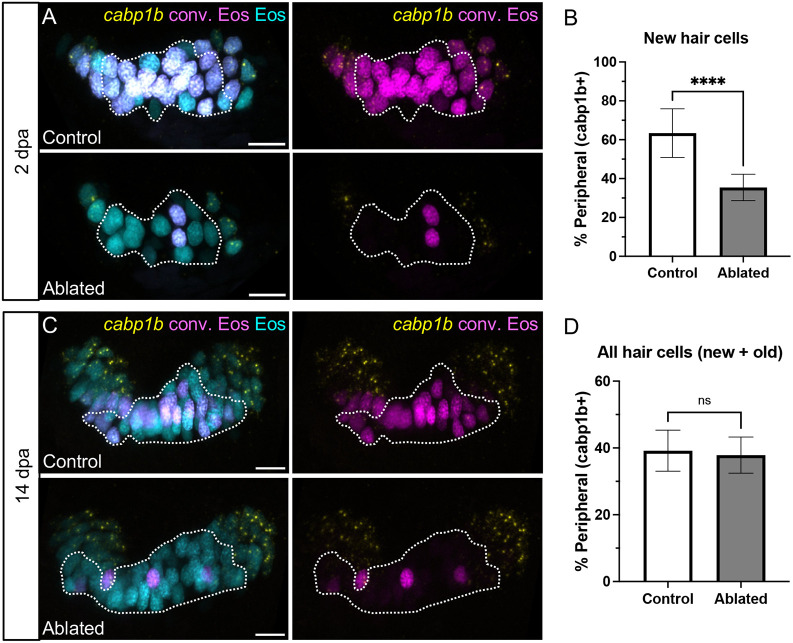
**Hair cell central-peripheral patterning is restored after ablation.** (A) Representative maximum intensity projections of anterior crista in control and ablated fish at 2 dpa with *cabp1b* HCR-FISH. Photoconverted Eos (magenta) and *cabp1b* (yellow) channels are shown with and without unconverted Eos (cyan). Dotted outline delineates the central *cabp1b*^−^ region of the sensory patch. (B) Quantification of *cabp1b*^+^ new hair cells, shown as a percentage of all new (cyan only) hair cells in control (*n*=18) and ablated (*n*=16) anterior cristae. Unpaired *t*-test, ****P<0.0001. (C,D) Analogous data to A,B for crista at 14 dpa (*n*=18 control, 15 ablated). Unpaired *t*-test, *P*=0.5226. Scale bars: 10 µm. Data are mean±s.d.

We next determined whether hair cells regenerated with appropriate spatial identity. We again used HCR probes against *cabp1b* to distinguish peripheral from central type regenerated hair cells. At 2 days post-ablation, newly added *cabp1b*^+^ and *cabp1b*^−^ hair cells were present in control and ablated conditions (Fig. 7A). In regenerating crista, the percentage of new hair cells of the *cabp1b*^+^ peripheral type was significantly decreased compared with controls (Fig. 7B). This suggests that the proportion of newly added central-type cells increases in the aftermath of hair cell ablation. To confirm this, we repeated this experiment using HCR probes for *scn5lab*, a marker of central crista hair cells (Fig. S7A). As expected, the proportion of new *scn5lab*^+^ central-type cells was significantly increased compared with controls (Fig. S7B). To determine whether organ patterning returned to that of homeostatic conditions after ablation, we probed for *cabp1b* in 14 dpa fish (Fig. 7C). At this timepoint, when total crista hair cell number in ablated fish had returned to control levels, the overall ratio of central to peripheral hair cells with their regular spatial patterning was also restored (Fig. 7D). Together, these data suggest that a memory of organ patterning and corresponding hair cell identities is maintained in cristae even after extensive hair cell loss.

### Hair cells regenerate primarily by transdifferentiation

**Fig. 8.**
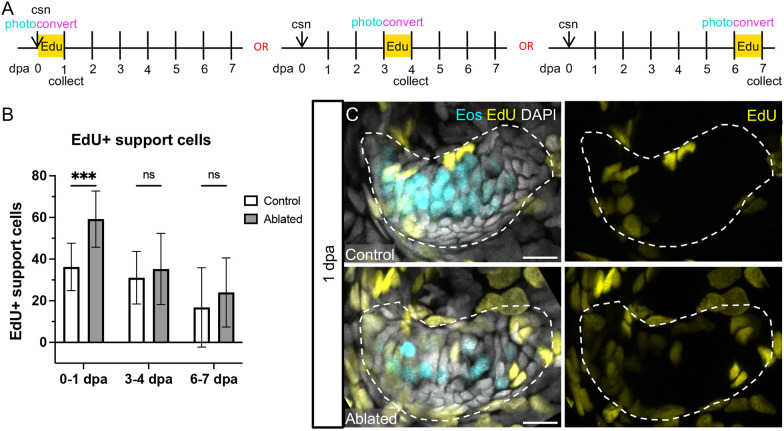
**Support cells proliferate in response to hair cell ablation.** (A) Larvae were incubated in EdU for 24 h immediately after hair cell ablation, at 3 dpa or at 6 dpa, and were collected at the end of the 24 h incubation. Photoconversion was performed before administration of EdU. (B) Quantification of EdU-labeled support cells in the combined anterior and lateral cristae in control and ablated fish incubated in EdU at 0-1 dpa (*n*=13 control, 14 ablated), 3-4 dpa (*n*=19, 12) or 6-7 dpa (*n*=9, 7). Two-way ANOVA is significant across condition (*P*=0.0021). Šídák's multiple comparisons post-hoc test at 0-1 dpa, ***adjusted *P*-value=0.0004. Data are mean±s.d. (C) Representative maximum intensity projections of anterior crista in control and ablated fish incubated with EdU at 0-1 dpa with Eos-labeled hair cells in cyan and EdU-labeled nuclei in yellow. Scale bars: 10 µm.

To determine whether proliferative mechanisms are used to regenerate hair cells in the zebrafish inner ear, we applied EdU, a thymidine analog that incorporates into the DNA of dividing cells, resulting in labeled daughter nuclei (Salic and Mitchison, 2008). We performed 24 h EdU pulses in regenerating fish at 0-1 dpa, at 3-4 dpa and at 6-7 dpa (Fig. 8A, Fig. S8). Photoconversion was performed immediately before EdU treatment to identify hair cells added during the EdU pulse. At 1 dpa, EdU-labeled hair cells in both control and ablated conditions were rare, less than 1% (Table 1), suggesting that the vast majority of hair cells added immediately post-ablation do not arise from recently dividing progenitors. Owing to the rarity of EdU labeled hair cells in individual cristae, cell counts for the anterior and lateral cristae were combined for these analyses. In both conditions, in cases where rare EdU^+^ hair cells were observed, they were found paired with an EdU^+^ supporting cell (Fig. S9), suggesting that a low level of asymmetric division may occur. There was no change at either 4 or 7 dpa, with EdU-labeled hair cells still rare (Fig. S8, Table 1), indicating that a later wave of proliferative hair cell regeneration did not occur. We conclude that transdifferentiation is the predominant mechanism by which hair cells are added to regenerating cristae.

**Table 1.**
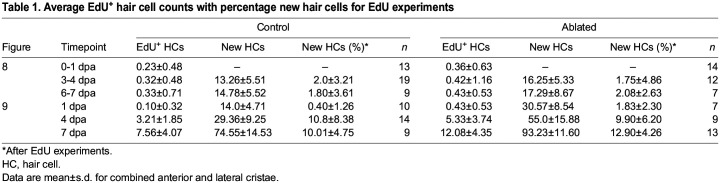
Average EdU^+^ hair cell counts with percentage new hair cells for EdU experiments

### Hair cell ablation leads to temporary expansion of supporting cells

In contrast to hair cells, EdU-labeled nonsensory cells were common after the 0-1 dpa EdU pulse. When located near hair cells, as opposed to around the periphery of the sensory organ, these EdU^+^ cells expressed the support cell marker *zpld1a* by HCR (Fig. S10). Significantly more EdU-labeled supporting cells were present in ablated cristae compared with controls at 1 dpa (Fig. 8B,C). However, there was no significant difference in the number of EdU-labeled supporting cells between control and ablated fish after the 3-4 dpa or 6-7 dpa EdU pulses (Fig. S8). These results demonstrate that there is an early wave of supporting cell proliferation in response to hair cell damage that is not sustained at later periods.

**Fig. 9.**
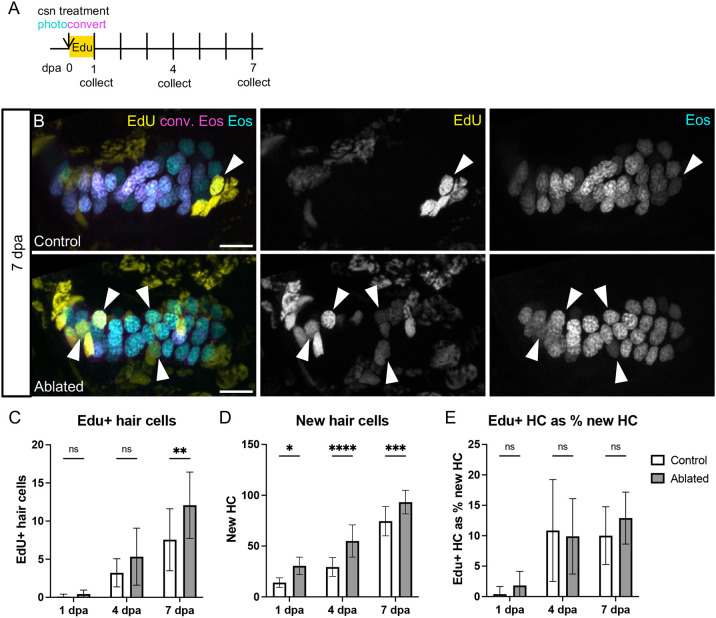
**EdU-labeling of hair cells over the week after ablation.** (A) Larvae were incubated in EdU for 24 h after photoconversion and hair cell ablation, and were collected either at the end of the incubation (1 dpa; *n*=10 control, 7 ablated) or at 4 (*n*=14, 8) or 7 (*n*=9, 13) dpa. (B) Representative maximum intensity projections of anterior crista in control and ablated fish at 7 dpa. White arrowheads indicate examples of hair cells added since ablation with EdU signal (yellow) and only unconverted Eos (cyan, no magenta). Scale bars: 10 µm. (C) Quantification of EdU^+^ hair cells in the combined anterior and lateral cristae at each timepoint in control and ablated fish. Two-way ANOVA is significant across conditions, *P*=0.0050; Šídák's multiple comparisons post-hoc test 7 dpa, **adjusted *P*-value=0.0034. (D) Quantification of new (cyan only) hair cells at each timepoint in control and ablated fish. Two-way ANOVA is significant across conditions, *P*<0.0001; Šídák's multiple comparisons post-hoc test at 1 dpa, *adjusted *P*-value=0.0123, at 4 dpa, ****adjusted *P*-value<0.0001, and at 7 dpa, ***adjusted *P*-value=0.0010. (E) EdU^+^ hair cells as a percentage of new hair cells. Two-way ANOVA with Šídák's multiple comparisons post-hoc test is not significant across conditions at any timepoint. Data are mean±s.d.

To determine whether supporting cells that divide in response to hair cell ablation ultimately become hair cells, we repeated the regeneration experiment with an EdU pulse during the first 24 h of regeneration and collected fish at 1, 4 or 7 dpa (Fig. 9A, Fig. S11). Again, we observed a significant increase in EdU-labeled supporting cells at 1 dpa compared with controls (Fig. S12), and rare labeled hair cells in both control and ablated conditions (Table 1). Although significantly elevated at 1 dpa, the number of EdU labeled supporting cells in ablated cristae returned to the level of controls by 4 dpa (Fig. S12). EdU-labeled hair cells were increasingly common at the 4 and 7 dpa timepoints in both control and ablated fish (Fig. 9B,C, Table 1). By 7 dpa, significantly more EdU-labeled hair cells were present in ablated crista (Fig. 9B,C), corresponding to the increase in supporting cells labeled at 1 dpa. The total number of new hair cells also significantly increased in ablated compared with control fish (Fig. 9D). When viewed as a percentage of all new hair cells, the fraction of EdU^+^ hair cells is not significantly different between ablated and control conditions at any timepoint (Fig. 9E). Therefore, supporting cells that divided in response to hair cell ablation are not more likely to differentiate into hair cells. These results suggest that, in the wake of hair cell ablation, supporting cells proliferate to increase the progenitor pool, but that this proliferative response is not coupled to the rate of hair cell differentiation.

## DISCUSSION

We describe a steady increase in hair cell number during the growth of inner ear sensory patches during the larval phase of zebrafish development, an approximately 1 month-long period after embryogenesis is complete. We used photoconvertible nuclear-localized Eos to distinguish pre-existing hair cells from newly added hair cells. We found that central and peripheral hair cell subtypes are added at the edges of the organ in a stereotyped pattern based on their location. We document a phenotypic switch of some older hair cells from peripheral to central subtype, resulting in conservation of spatial patterning and an overall ratio that slightly favors central-type hair cells. We also found that the number of photoconverted cells in cristae and utricle did not significantly decrease over time, suggesting that there is little hair cell turnover during larval stages.

We provide several lines of evidence that the addition of crista hair cells after damage is more than simply recovery by continued growth. We demonstrate, using photoconversion to parse the timing of differentiation, that new hair cells are added at a faster rate after hair cell ablation than during growth. We also found that compared with growth there was an increase in new hair cells of the central subtype, and as a result the organ regenerates the appropriate ratio of subtypes for correct spatial patterning. Finally, there is an increase in supporting cell proliferation in response to hair cell ablation, eventually resulting in more EdU-labeled hair cells than under control conditions. If proliferation and hair cell differentiation were directly coupled, we would expect to see a disproportionate number of dividing support cells become new hair cells. The lack of difference between the fraction of EdU-labeled new hair cells in control and ablated conditions indicates that the supporting cells dividing in response to ablation are no more likely than others to differentiate into new hair cells. Indeed, our experiments suggest that, during growth, supporting cells convert to hair cells using mechanisms temporally uncoupled from cell division, and regenerating hair cells are added through a similar process of transdifferentiation (Fig. S13). We hypothesize that the primary regenerative response to damage is to increase the pool of supporting cells available for differentiation into hair cells, employing the same conversion mechanisms used in normal growth to add new hair cells. Of particular note, the transient increase in supporting cell proliferation occurs before hair cell replacement, suggesting that the cue for this event is the damage or loss of hair cells rather than depletion of supporting cells through transdifferentiation. The relationship between supporting cell proliferation and hair cell differentiation could be further tested by assessing the effects of blocking proliferation on regeneration.

Although our current work examines hair cell regeneration in the larval zebrafish cristae over the first month of development, our findings are consistent with previous studies examining regeneration in the zebrafish maculae. Lineage tracing in the embryonic utricle after laser ablation of hair cells provides evidence that supporting cells directly transdifferentiate into nascent hair cells (Millimaki et al., 2010). In the adult saccule, noise damage induces a burst of proliferation that occurs 1-3 days post sound exposure with regenerated hair cell bundles formed in the most damaged area of the organ over approximately 10 days (Schuck and Smith, 2009), a timeline that is consistent with our findings in the cristae. Single-cell RNA-sequencing data from regenerating maculae of adult zebrafish point to the emergence of a transition-state population with qualities of both hair and supporting cells, which could potentially represent actively transdifferentiating cells (Jimenez et al., 2022). Together, these studies support a model where damage induces hair cell regeneration through transdifferentiation and expansion of supporting cells through proliferation.

Our findings in the zebrafish inner ear are markedly different from the mechanism of regeneration observed in the zebrafish lateral line system. After hair cell ablation by ototoxic drug exposure, neuromasts show significant hair cell replacement after 24 h and regenerate a full complement of hair cells in only 72 h (Ma et al., 2008), compared with a gradual replacement of hair cells that we observe in the cristae over the course of 2 weeks. Lateral line hair cells are regenerated in pairs by symmetrically dividing precursors (Lopez-Schier and Hudspeth, 2006; Mackenzie and Raible, 2012; Romero-Carvajal et al., 2015; Wibowo et al., 2011), while we find those in the cristae are overwhelmingly added by transdifferentiation. The rare examples of EdU-labeled hair cells we observed in the cristae were adjacent to labeled supporting cells, suggesting asymmetric division of precursors. We speculate these differences may reflect the need of the lateral line system to restore the integrity of organs exposed to the environment on the surface of the fish, while regeneration in the inner ear occurs on top of extensive growth and is needed to restore appropriate spatial patterning in addition to organ integrity.

Comparison of growth and regeneration in the inner ear of zebrafish with that in birds reveals both similarities and differences. Regeneration of hair cells in avian auditory and vestibular systems occurs by both transdifferentiation and proliferative replacement. In the regenerating avian utricle, there is evidence that hair cells are replaced both by asymmetric divisions and by transdifferentiation (Scheibinger et al., 2022; Stone et al., 1999). When hair cells are regenerated in the auditory organ, the basilar papilla (BP), they are initially added by wave of transdifferentiation that lasts for several days before a second phase of proliferative hair cell regeneration begins (Roberson et al., 1996, 2004). To determine whether there is a similar late wave of proliferation in the zebrafish cristae, we administered pulses of EdU at timepoints several days after ablation but did not observe any increase in EdU-labeled hair or supporting cells compared with controls. Thus, in the zebrafish larval cristae, there appears to be a single mechanism of transdifferentiation for hair cell replacement. In the mature avian vestibule, there is significant hair cell turnover, with hair cells having an estimated half-life of about 20-30 days as they are removed and replaced via asymmetric division (Goodyear et al., 1999; Jørgensen and Mathiesen, 1988; Kil et al., 1997; Weisleder and Rubel, 1992). We have observed no evidence for turnover in the zebrafish cristae during larval stages but cannot rule out the possibility of rare events or turnover at later stages. In the few cases where we observed hair cells labeled by EdU, they were accompanied by a neighboring Edu-labeled supporting cell, suggesting that a small amount of asymmetric division may also occur in the zebrafish inner ear.

Our findings show remarkable similarities to processes that occur in the mammalian vestibular system (Burns et al., 2012a; Wang et al., 2015). When damage is induced in the utricle of neonatal mice, new hair cells are initially generated by transdifferentiation of supporting cells, with an accompanying wave of supporting cell proliferation detected by EdU incorporation. In the following weeks, a fraction of these EdU-labeled cells becomes new hair cells. However, the regenerative response is greatly diminished after the first week postpartum. These regenerative events parallel processes that occur during the normal postnatal growth of the mouse utricle, where approximately half of hair cells are added over the 3 weeks after birth from supporting cells that last divided before birth (Burns et al., 2012b). In adult mice, limited regeneration occurs by transdifferentiation of supporting cells with no detected proliferative response for their replacement, and as a consequence an overall reduction in supporting cell numbers is observed (Golub et al., 2012). Hair cell turnover, although detectable in the adult mouse utricle, is rare and not associated with supporting cell proliferation (Bucks et al., 2017). Taken together, these studies support the idea that there is uncoupled potential for both proliferative and transdifferentiation responses in the mouse utricle that wane over time.

Our study establishes the zebrafish inner ear as a model for hair cell regeneration that parallels processes that are functional for a limited period in mammals. A major difference between mammals and zebrafish is that they lose their ability to functionally regenerate in response to damage (Burns et al., 2012a; Cox et al., 2014), even in response to exogenous factors such as altering Notch signaling or inducing Atoh1 expression (Liu et al., 2012; Maass et al., 2015). Whether mammals lose their ability to regenerate due to epigenetic changes affecting chromatin accessibility (Tao et al., 2021), to alterations in cell cycle regulation (White et al., 2006), to changes in tissue architecture (Burns and Corwin, 2014; Collado et al., 2011) or to a combination with other unknown factors remains an area of active study.

Zebrafish have a remarkable ability to regenerate many organs, including the heart, liver, kidney, fin, retina and central nervous system (reviewed by Marques et al., 2019), some of which show similarities to the inner ear regeneration mechanism we describe here. In the zebrafish olfactory bulb, death of sensory neurons by chemical exposure results in proliferation of the precursor pool during the first 24 h after neuron death (Ma et al., 2018). Transdifferentiation has been observed during regeneration of other zebrafish organ systems. After major damage to the liver, biliary epithelial cells proliferate and transdifferentiate into regenerated hepatocytes (Choi et al., 2014). In the pancreas, upon ablation of insulin-responsive β-cells, some α-cells transdifferentiate into β-cells whereas others respond by proliferating, presumably to replace converting α-cells (Ye et al., 2015). Other organs do not exhibit transdifferentiation but rely on a resident population of multipotent cells that act in growth and regeneration. As in the ear and other organs, zebrafish kidneys grow throughout life in proportion to fish size (Zhou et al., 2010). Some ototoxic drugs, such as aminoglycoside antibiotics, also demonstrate nephrotoxicity. After injection of the aminoglycoside gentamicin, adult zebrafish regenerate nephrons over the course of 2 weeks (Diep et al., 2011). In this case, regeneration is facilitated by a resident stem cell population that acts both in adult nephrogenesis as well as regeneration (Diep et al., 2011). In the adult zebrafish central nervous system, the telencephalon contains radial glia that proliferate under homeostatic conditions (Rothenaigner et al., 2011). These same glia respond to lesion injury with proliferation and give rise to neuroblasts that migrate to the site of injury, where they differentiate into mature neurons (Kroehne et al., 2011). Our work indicates that support cells of the inner ear may represent a similar resident facultative progenitor population that can self-renew and generate hair cells during growth and regeneration. Whether inner ear support cells comprise subpopulations with differential potential to give rise to hair cells remains an unanswered question.

## MATERIALS AND METHODS

### Fish maintenance

Experiments were conducted on larval zebrafish between 5 dpf and approximately 45 dpf (up to 11.0 mm SL). Larvae were raised in E3 embryo medium (14.97 mM NaCl, 500 mM KCl, 42 mM Na_2_HPO_4_, 150 mM KH_2_PO_4_, 1 mM CaCl_2_ dihydrate, 1 mM MgSO_4_ and 0.714 mM NaHCO_3_ at pH 7.2) at 28.5°C and placed on the nursery system at 5 dpf. During experiments, larval fish were returned to the nursery system between treatment and collection timepoints, except during EdU incubation or when collected immediately after treatment. Zebrafish experiments and husbandry followed standard protocols in accordance with the University of Washington Institutional Animal Care and Use Committee guidelines.

### Transgenic lines

The *Tg(myo6b:TrpV1-mClover)^w273Tg^* was constructed using rat TrpV1 (Chen et al., 2016) fused to the fluorescent reporter mClover3 (Bajar et al., 2016) under the control of the zebrafish *myo6b* promoter (Kindt et al., 2012). The *Tg(myo6b:nls-Eos)^w191Tg^* line has been described previously (Cruz et al., 2015). All transgenic fish lines were crossed into a *nac/roy* background (*mitfa^w2^; mpv17^a9^*; Lister et al., 1999; Ren et al., 2002; White et al., 2008) to facilitate inner ear imaging.

### Photoconversion

Larvae were transferred to a 60×15 mm petri dish and placed in a freezer box lined with aluminum foil. An iLumen 8 UV flashlight (procured from Amazon) was fixed to the freezer box lid and positioned over the dish. Larvae were exposed to UV light for 10 min before being returned to standard 100×15 mm petri dishes to await experimentation.

### TrpV1 hair cell ablation

Capsaicin (Sigma-Aldrich, M2028) was resuspended in DMSO and stored at −20°C until use. Dose-response curves were performed on *Tg(myo6b:TrpV1-mClover)* in both *AB and *nac/roy* backgrounds. There were no apparent differences in response to capsaicin treatment between fish of the two backgrounds. Capsaicin (10 μM) was determined to be an appropriate dose to effectively ablate cristae hair cells when treated for 1 h at 28.5°C. The brightness of Eos in the *Tg(myo6b:nls-Eos)* line prevents normal fluorescent dissecting scope screening for *Tg(myo6b:TrpV1-mClover)*, even after Eos has been photoconverted. 8 dpf *Tg(myo6b:nls-Eos)* siblings with and without *Tg(myo6b:TrpV1-mClover)* were treated with 10 μM capsaicin for 1 h at 28.5°C. Larvae were washed three times for 5 min each in system water. Larvae were then screened for dying hair cells to indicate the presence (ablated) or absence (control) of *TrpV1-mClover*. Ablated and control fish were separately returned to the nursery system to await collection.

### EdU treatment and visualization

Larvae were incubated in 500 μM F-ara-EdU (Sigma, T511293) for 24 h at 28.5°C. The Click-iT protocol was modified from Salic and Mitchison (2008). Briefly, larvae were fixed in 4% paraformaldehyde at 4°C for 18-48 h, depending on their size, then washed with PBS containing 0.1% Tween20 three times for 10 min each. Larvae were permeabilized in 0.5% TritonX-100 in PBS for 30 min and washed three times for 10 min each with PBS alone. A reaction solution was prepared fresh each time: 2 mM CuSO_4_, 10 mM Alexa Fluor 647 azide and 20 mM sodium ascorbate in PBS. Fish were incubated in reaction solution for 1 h in the dark at room temperature, washed three times for 20 min each with PBS, and stored in the dark at 4°C until imaging.

### HCR FISH

Hybridization chain reaction *in situ* hybridizations (Molecular Instruments, HCR v3.0) were performed as directed for whole-mount zebrafish embryos and larvae (Choi et al., 2016, 2018). Briefly, larvae were fixed in 4% PFA at 4°C for 18-48 h. Larvae were washed with PBS and transferred to methanol to be stored at −20°C until use. Larvae were rehydrated using a gradation of methanol and PBST washes, treated with proteinase K for 25 min and post-fixed with 4% PFA for 20 min at room temperature. For the detection phase, larvae were pre-hybridized with a probe hybridization buffer for 30 min at 37°C, then incubated with probes overnight at 37°C. Larvae were washed with 5×SSCT to remove excess probes. For the amplification stage, larvae were pre-incubated with an amplification buffer for 30 min at room temperature and incubated with hairpins overnight in the dark at room temperature. Excess hair pins were removed by washing with 5×SSCT. Larvae were transferred to storage buffer and kept in the dark at 4°C until imaging.

### Fixation and imaging preparation

Larvae were fixed in 4% paraformaldehyde at 4°C for 18-48 h, depending on their size. Larvae were washed three times for 15 min each in PBS containing 0.1% Tween20 and transferred to storage buffer (PBS containing 0.2% Triton, 1% DMSO, 0.02% sodium azide and 0.2% BSA). Samples were stored for no more than 3 weeks at 4°C before imaging. Fixed fish were mounted by first drawing a thin ring of vacuum grease on the underside of a coverslip. One or more specimens were placed on their side in the center of the ring along with one or two drops of PBS or other storage solution. A second coverslip was placed on top and gently pushed down at the sides to create a seal around the samples to prevent evaporation and drifting while imaging. Coverslip ‘sandwiches’ were overlaid on a flat ruler under a dissecting microscope, and standard length for each fish was measured, estimating to the nearest 0.25 mm. Hair cells in the cristae and utricle were counted in fixed intact fish as much as possible. When larval fish grew beyond approximately 8 mm, it became necessary to dissect the ear in order to image and perform accurate hair cell counts.

### Imaging

Images for development, turnover and regeneration timeline experiments were captured on a Zeiss LSM-880 with Airyscan 1.0 functionality. *Z*-stacks of inner ear organs were taken using a 20×/0.8 air objective at intervals of 0.32 μm. Development, turnover, regeneration timeline, EdU, and HCR experimental images were captured using a Zeiss LSM-980 with Airyscan 2.0. *Z*-stacks of inner ear organs were taken using a 25×/0.8 water objective at intervals of 0.58 μm. *Z*-stacks of whole ears were taken using a 10×/0.45 air objective at intervals of 1.32 μm. All Airyscan processing was performed at standard strength using Zen Blue software (Zeiss, www.zeiss.com). Image processing and data analysis were carried out using Fiji (Schindelin et al., 2012).

### Statistical analysis

Power analyses were carried out in G*Power (Faul et al., 2007) using preliminary data to determine sample sizes. All other statistical analyses were performed in GraphPad Prism version 10.1.0 (www.graphpad.com).

## Supplementary Material



10.1242/develop.202944_sup1 Supplementary information
