# Supplementary material for: Transdifferentiation is temporally uncoupled from progenitor pool expansion during hair cell regeneration in the zebrafish inner ear
Source: Development. 2024 Aug 13;151(15):dev202944. doi: 10.1242/dev.202944 (PMC11361639; doi:10.1242/dev.202944)
Supplement: Supplementary information [file develop-151-202944-s1.pdf]

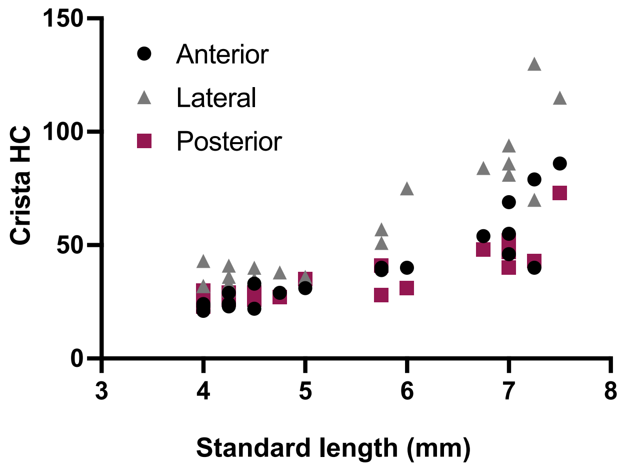

Fig. S1. The posterior crista is similar in size to the anterior crista

Hair cell counts from the anterior, lateral, and posterior cristae from the same set of fish. Each data point represents one ear from one fish (n = 21).

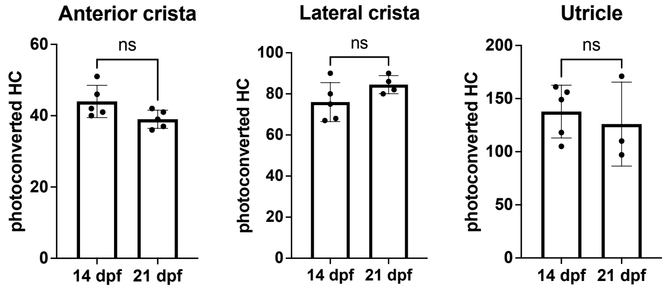

Fig. S2. Little hair cell turnover occurs in the third week post-fertilization

Quantification of anterior crista, lateral crista, and utricle photoconverted hair cells at 14 (n = 5 ant crista, 5 lat crista, 5 utricle) and 21 dpf (n = 5 ant crista, 4 lat crista, 3 utricle). Mann-Whitney tests indicate no significant difference between the number of photoconverted hair cells at these two timepoints (ant crista p = 0.095, lat crista p = 0.174, utricle p = 0.786). All data is presented as mean ± s.d.

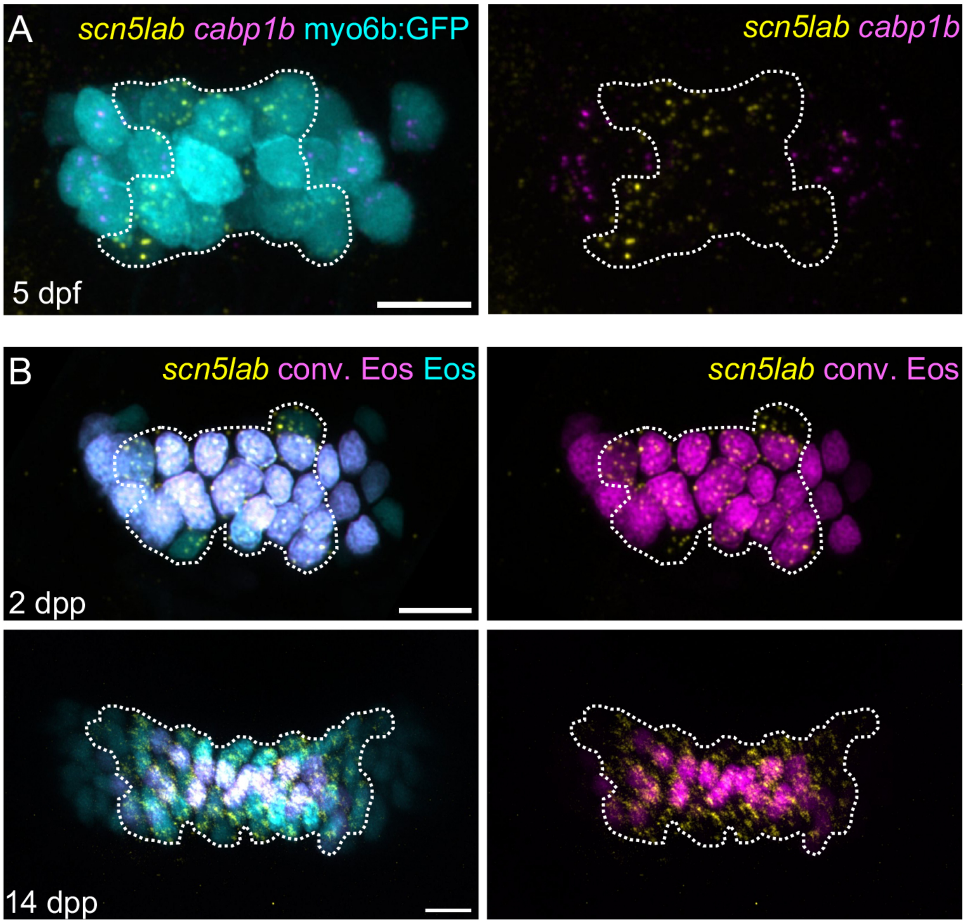

Fig. S3. Crista growth with scn5lab HCR

A) Representative maximum intensity projection of the anterior crista of *Tg(myo6b:GFP)* (cyan) 5 dpf larvae treated with HCR *cabp1b* (magenta) and *scn5lab* (yellow) probes to label peripheral- and central-type hair cells, respectively. Dotted outline delineates central, *cabp1b*-;*scn5lab*+ region of the sensory patch. B) Representative maximum intensity projections of anterior crista 2 dpp and 14dpp with *scn5lab* HCR-FISH. Photoconverted Eos (magenta) and *scn5lab* (yellow) channels are shown with and without unconverted Eos (cyan). Dotted outline delineates central, *scn5lab*+ region of the sensory patch. Scale bars = 10 μm

Development • Supplementary information

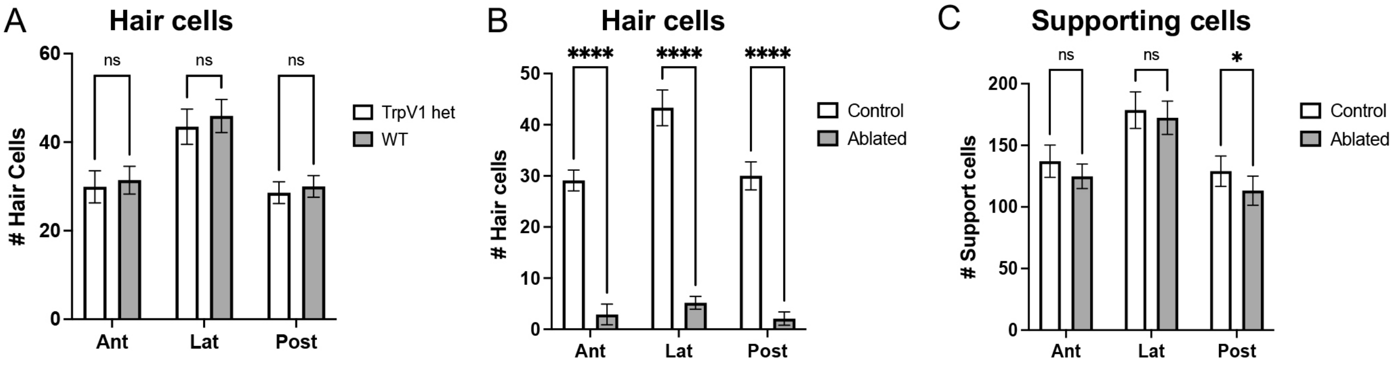

Fig. S4. Hair and supporting cell counts in fish with and without *Tg(myo6b:TrpV1-mClover)*

A) Quantification of hair cells in each crista of wild type (n = 12) and *Tg(myo6b:TrpV1-mClover)* heterozygous (n = 12) fish at 8dpf. Two-way ANOVA with Šídák's multiple comparisons post-hoc test indicates no significant differences across condition in any crista. B) Quantification of hair cells in each crista of control (*Tg(myo6b:NLS-Eos)*, n = 10) and ablated (*Tg(myo6b:NLS-Eos)*; *Tg(myo6b:TrpV1-mClover)*, n = 11) heterozygous fish after capsaicin treatment at 8dpf. Two-way ANOVA for hair cell counts with Šídák's multiple comparisons post-hoc test is significant across condition p < 0.0001, Šídák's multiple comparisons post-hoc test for all three crista adjusted p-value < 0.0001. C) Quantification of supporting cells from same fish as in panel B. Two-way ANOVA for supporting cell counts is significant across condition p = 0.0006, Šídák's multiple comparisons post-hoc test posterior crista adjusted p-value = 0.0167. All data is presented as mean ± s.d.

Development • Supplementary information

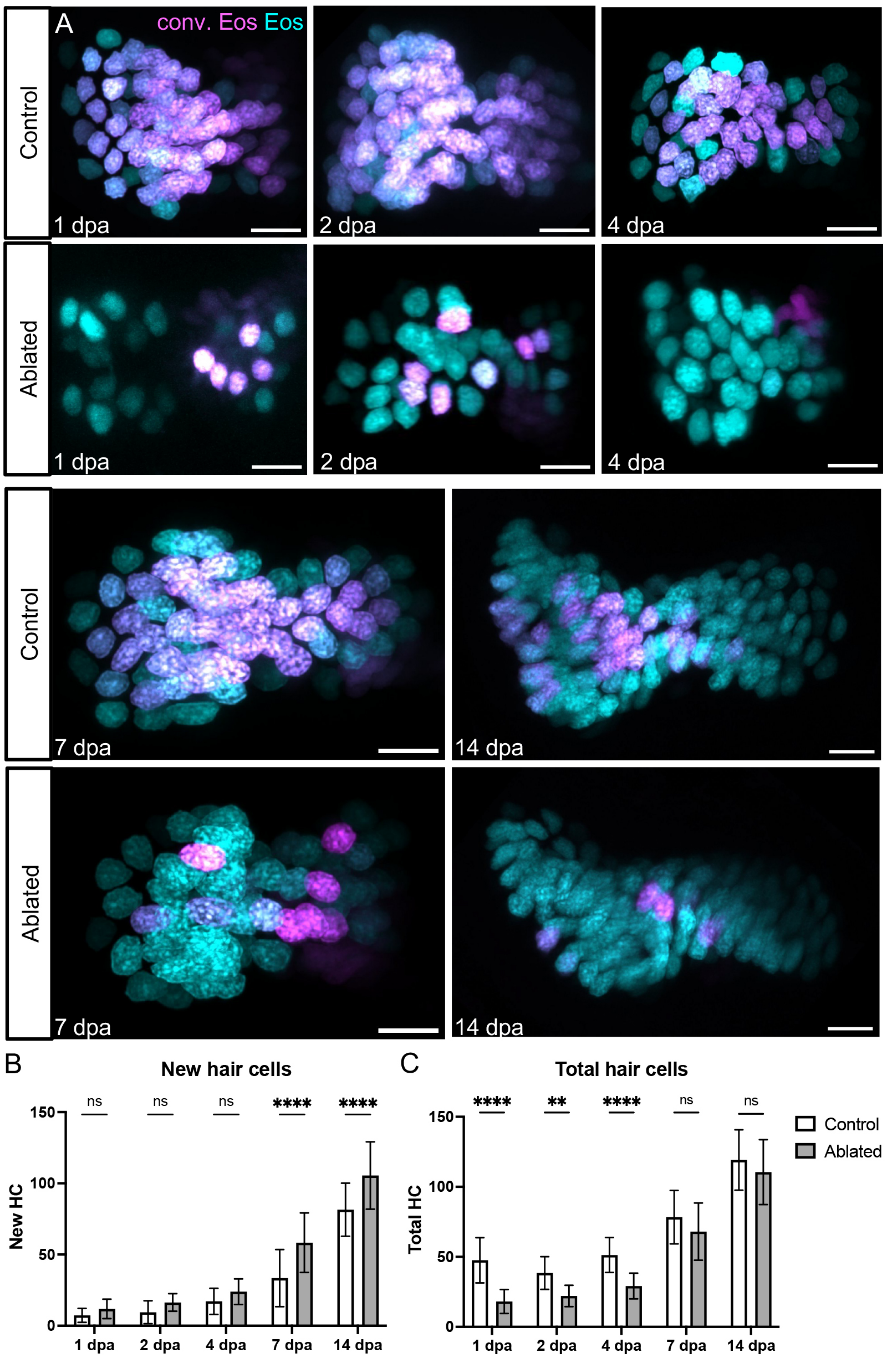

Fig. S5. Lateral crista hair cells regenerate during the two weeks following ablation
A) *Tg(myo6b:NLS-Eos)* sibling larvae with or without *Tg(myo6b:TrpV1-mClover)* were photoconverted and treated with capsaicin to ablate hair cells at 8dpf. Larvae were collected at five timepoints over the following two weeks: 1 (n = 22 control, 25 ablated), 2 (n = 20, 22), 4 (n = 19, 17), 7 (n = 16, 12), or 14 (n = 18, 14) days post-ablation (dpa). Representative maximum intensity projections of lateral crista in control and ablated fish at five timepoints following treatment. Nuclei of cells that survived capsaicin treatment contain photoconverted

Eos (magenta). Hair cells newly added after capsaicin treatment have nuclei with only unconverted Eos (cyan). Scale bars = 10 µm. B) Quantification of new (cyan-only) hair cells in ablated and control lateral crista. Two-way ANOVA variation across condition $p < 0.0001$; Šídák's multiple comparisons post-hoc test for 7 dpa adjusted p-value < 0.0001, 14 dpa adjusted p-value < 0.0001. C) Quantification of total hair cells in ablated and control anterior crista. Two-way ANOVA variation across condition $p < 0.0001$; Šídák's multiple comparisons post-hoc test for 1 dpa adjusted p-value < 0.0001, 2 dpa adjusted p-value = 0.0051, 4 dpa adjusted p-value < 0.0001. All data is presented as mean ± s.d.

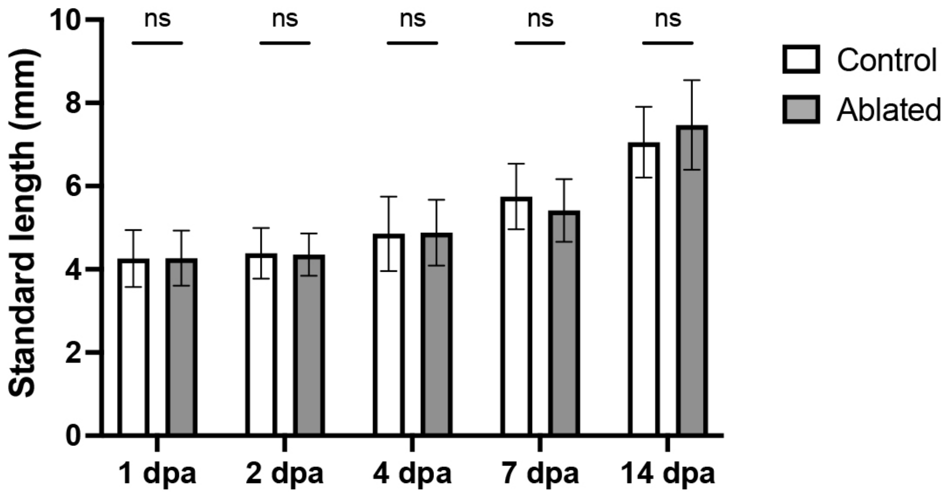

Fig. S6. Crista hair cell ablation does not affect larval growth

Standard length measurements across 1 (n = 23 control, 27 ablated), 2 (n = 13, 22), 4 (n = 12, 17), 7 (n = 15, 6), or 14 (n = 32, 16) dpa timepoints for ablated and control larvae. Two-way ANOVA with Šídák's multiple comparisons post-hoc test indicates no significant differences across condition at any timepoint.

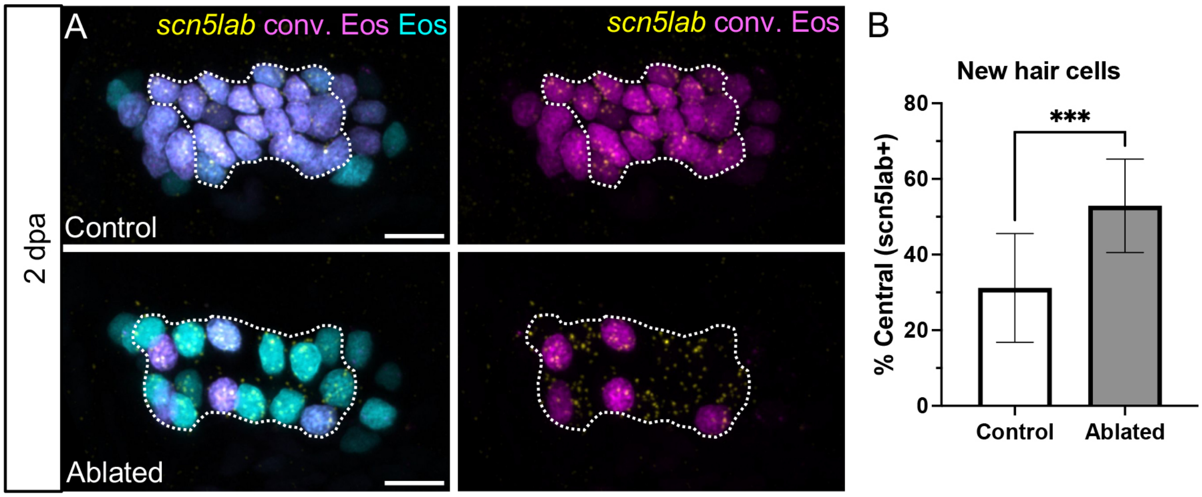

Fig. S7. Central-type hair cells are preferentially added following hair cell ablatio

A) Representative maximum intensity projections of anterior crista in control and ablated fish at 2 dpa with *scn5lab* HCR-FISH. Photoconverted Eos (magenta) and *scn5lab* (yellow) channels are shown with and without unconverted Eos (cyan). Dotted outline delineates central, *scn5lab*+ region of the sensory patch. Scale bars = 10 μm B) Quantification of *scn5lab*+ new hair cells, shown as a percentage of all new (cyan-only) hair cells in control (n = 13) and ablated (n = 17) anterior cristae. Unpaired t test p = 0.0001.

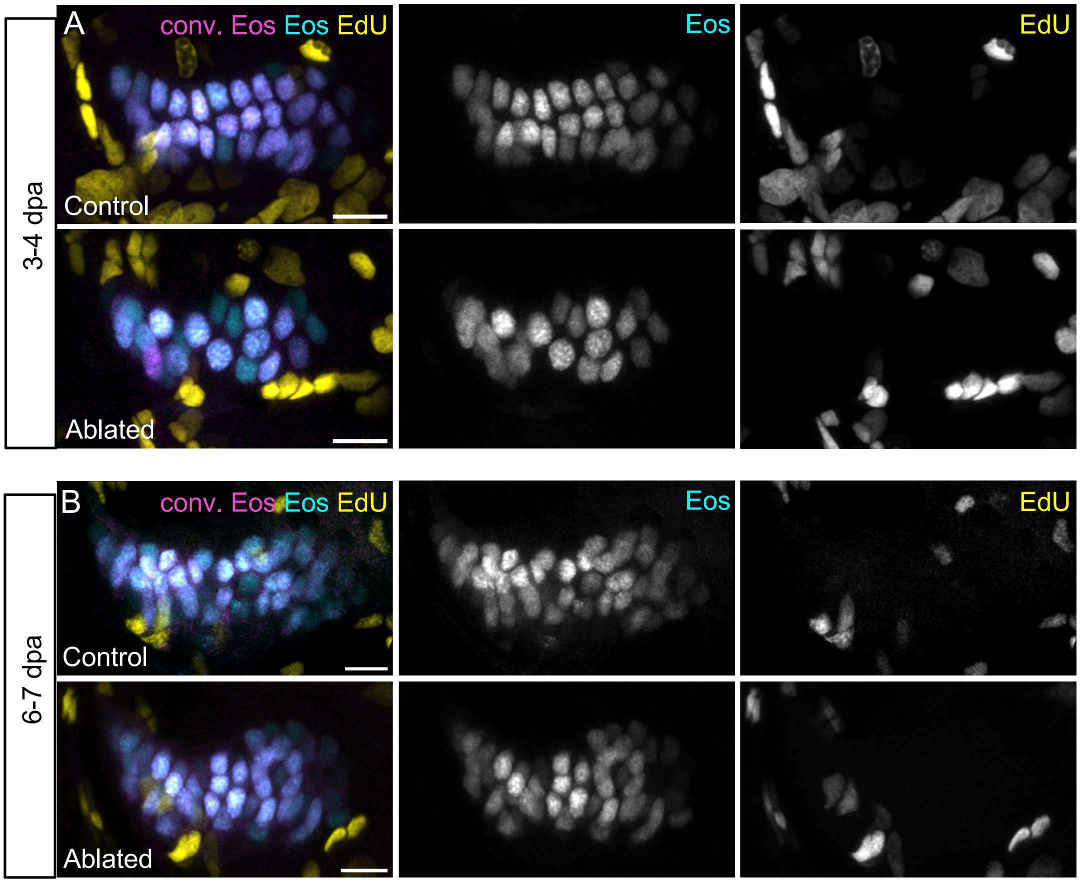

Fig. S8. Representative images from 3-4 and 6-7 day EdU pulses

Representative maximum intensity projections of anterior crista in control and ablated fish A) photoconverted at 3dpa and incubated with EdU from 3-4 dpa and B) photoconverted at 6dpa and incubated with EdU from 6- 7dpa. Hair cells added during EdU incubation have nuclei with unconverted Eos (cyan) and without converted Eos (magenta). EdU-labeled nuclei are shown in yellow. EdU+ hair cell nuclei were extremely rare – see Table 1 for quantification. Scale bars = 10 µm.

Development • Supplementary information

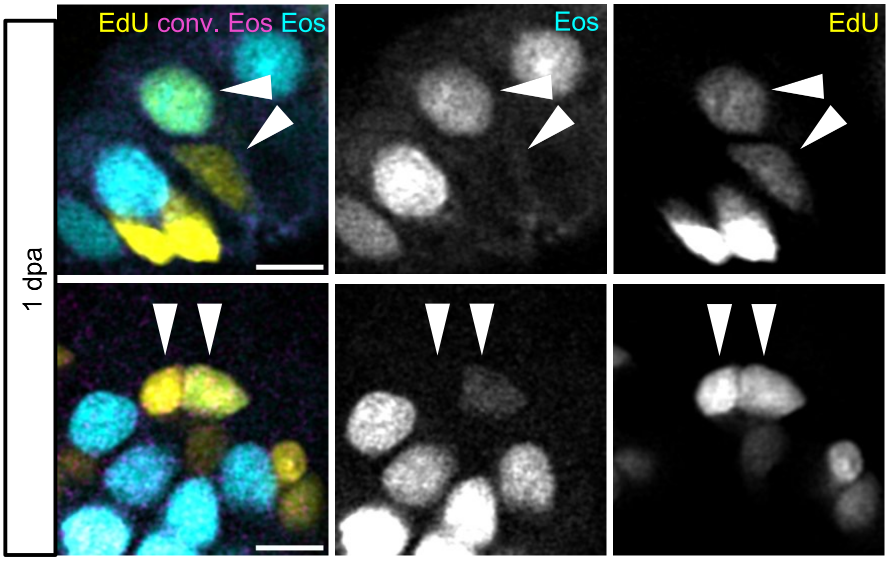

Fig. S9. EdU-labeled hair cell-supporting cell pairs are observed following hair cell ablation

Two examples of hair cell-supporting cell EdU+ pairs in ablated fish after 24h EdU incubation (1 dpa). Arrows indicate pairs where both cells are labeled with EdU (yellow), but only one expresses the Eos (cyan) hair cell marker. Scale bars = 5 µm

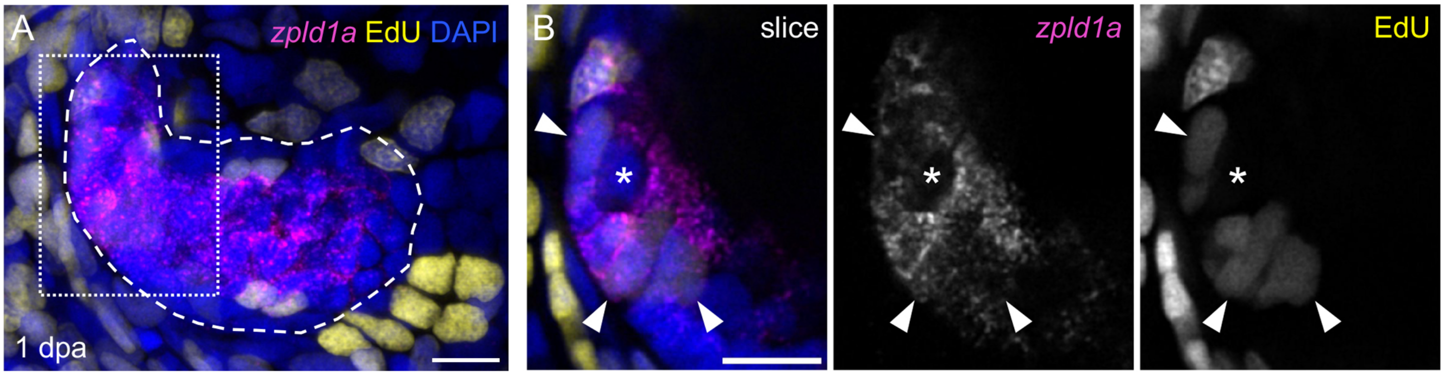

Fig. S10. EdU+ cells adjacent to HC are zpld1a+ support cells

A) Representative maximum intensity projection of the anterior crista from ablated fish incubated in EdU from 0-1dpa and collected at 1dpa with HCR for the crista support cell marker gene zpld1a. B) Individual slice of max projection inset shows EdU+ cells within the crista also express zpld1a. Arrowheads point to examples of EdU+ nuclei with zpld1a labeling. Star indicates a hair cell. Scale bars = 10 µm.

Development • Supplementary information

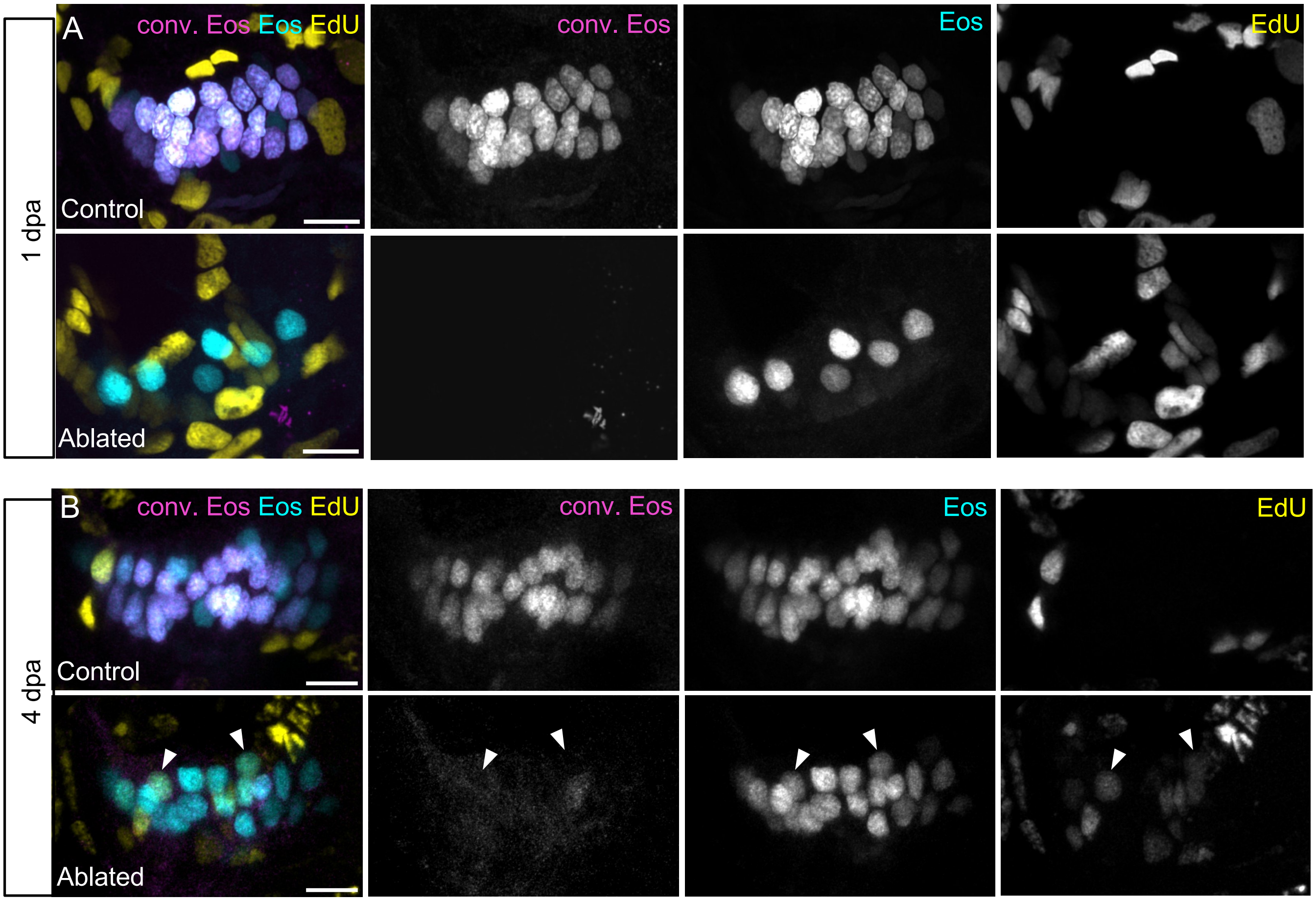

Fig. S11. Representative images from 0-1dpa EdU pulse collected at 1dpa and 4dpa

Representative maximum intensity projections of anterior crista in control and ablated fish incubated with EdU from 0-1 dpa and collected at A) 1dpa or B) 4 dpa. See Table 1 for quantification of Edu+ and new hair cell nuclei. White arrowheads indicate examples of hair cells added since ablation with EdU signal (yellow) and only unconverted Eos (cyan, no magenta). Scale bars = 10 μm.

Development • Supplementary information

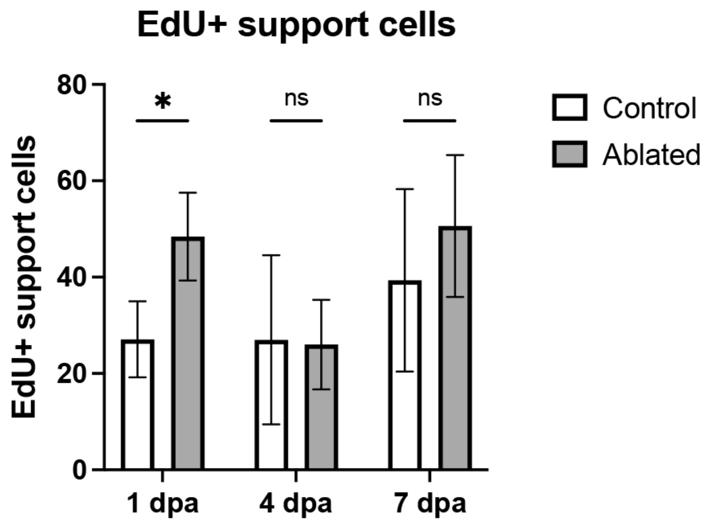

Fig. S12. EdU-labeling of supporting cells over the week following ablation

Larvae were incubated in EdU for 24 hours after photoconversion and hair cell ablation and collected either at the end of the incubation (1 dpa; n = 10 control, 7 ablated) or at 4 (n = 14, 8) or 7 (n = 9, 13) dpa. Quantification of EdU+ supporting cells in the combined anterior and lateral cristae at each timepoint in control and ablated fish. Two-way ANOVA is significant across condition p = 0.0069, Šídák's multiple comparisons post-hoc test 1 dpa adjusted p-value = 0.0107. All data is presented as mean ± s.d.

Development • Supplementary information

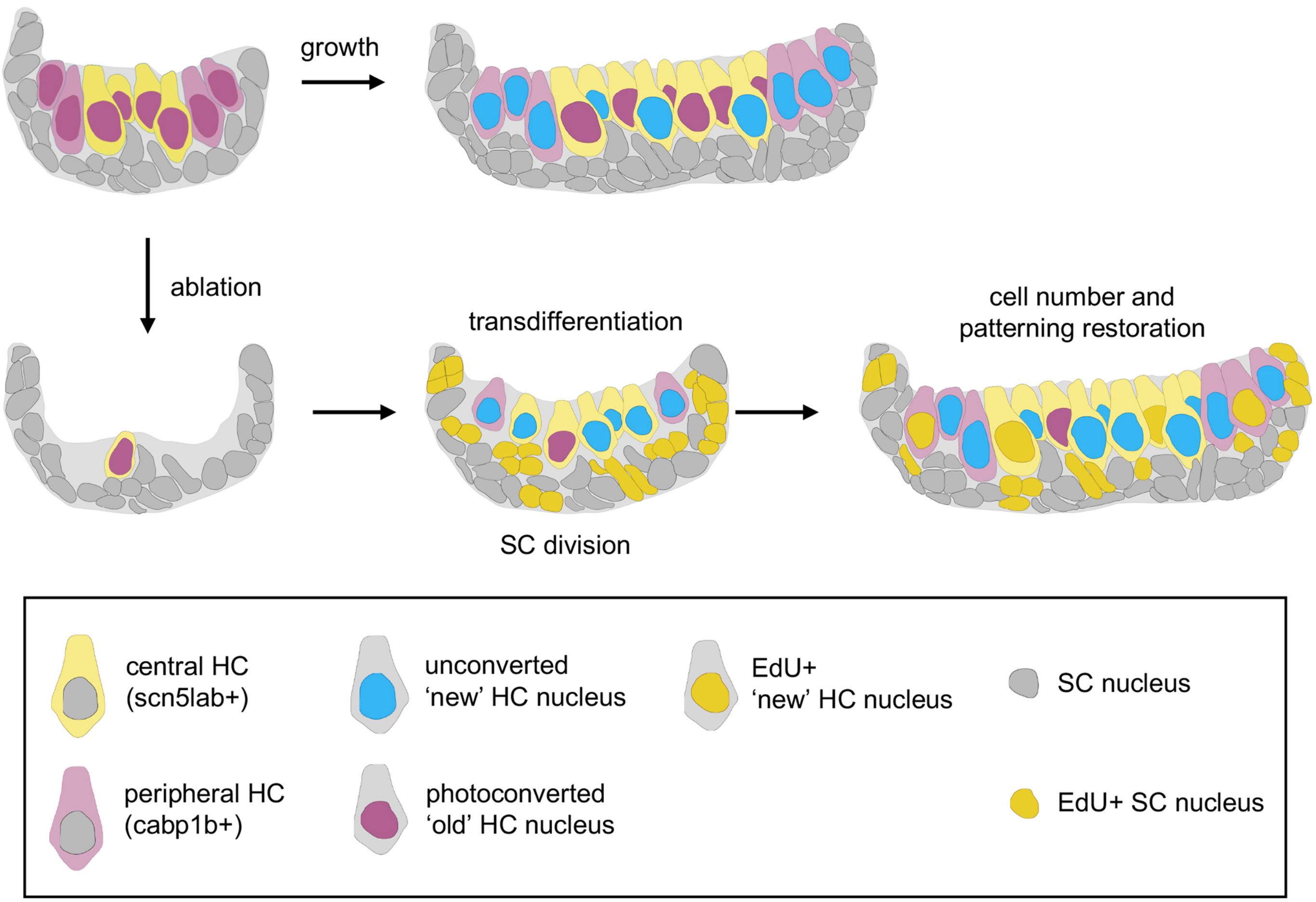

Fig. S13. Summary diagram

Diagrams depict a side-view cross section of a crista. Hair cells at the long edges of the organ express *cabp1b* and are 'peripheral' type, whereas hair cells in the middle region express *scn5lab* and are 'central' type. When the organ grows, new central and peripheral type cells are added. Hair cells that were peripheral type become more central within the organ and lose expression of *cabp1b*. When hair cells are ablated, supporting cell division increases. Hair cells are regenerated by direct transdifferentiation of supporting cells. The initial burst of supporting cell division is temporally uncoupled from hair cell replacement, which occurs gradually. The organ continues to grow during regeneration, and hair cell number and patterning are restored to normal by approximately two weeks after ablation.

Development • Supplementary information
