# Supplementary material for: Transdifferentiation is temporally uncoupled from progenitor pool expansion during hair cell regeneration in the zebrafish inner ear
Source: Development. 2024 Aug 13;151(15):dev202944. doi: 10.1242/dev.202944 (PMC11361639; doi:10.1242/dev.202944)
Supplement: Supplementary file 1 [file develop-151-202944_review_history.pdf]

Transdifferentiation is temporally uncoupled from progenitor pool expansion during hair cell regeneration in the zebrafish inner ear

Marielle O. Beaulieu, Eric D. Thomas and David W Raible
DOI: 10.1242/dev.202944

Editor: Kenneth Poss

Review timeline
Original submission 9 April 2024
Editorial decision: 20 May 2024
First revision received: 24 June 2024
Accepted: 15 July 2024

Original submission

First decision letter

MS ID#: DEVELOP/2024/202944

MS TITLE: Transdifferentiation is uncoupled from progenitor pool expansion during hair cell regeneration in the zebrafish inner ear

AUTHORS: Marielle O. Beaulieu, Eric D. Thomas, and David W Raible

I have now received all the referees' reports on the above manuscript, and have reached a decision. The referees' comments are appended below, or you can access them online: please go to BenchPress and click on the 'Manuscripts with Decisions' queue in the Author Area.

As you will see, each of the referees expresses considerable interest in your work, but have some criticisms and recommend a revision of your manuscript before we can consider publication. If you are able to revise the manuscript along the lines suggested, which may involve further experiments, I will be happy receive a revised version of the manuscript. Your revised paper will be re-reviewed by one or more of the original referees, and acceptance of your manuscript will depend on your addressing satisfactorily the reviewers' major concerns. Please also note that Development will normally permit only one round of major revision. If it would be helpful, you are welcome to contact us to discuss your revision in greater detail. Please send us a point-by-point response indicating your plans for addressing the referees' comments, and we will look over this and provide further guidance.

Please attend to all of the reviewers' comments and ensure that you clearly highlight all changes made in the revised manuscript. Please avoid using 'Tracked changes' in Word files as these are lost in PDF conversion. I should be grateful if you would also provide a point-by-point response detailing how you have dealt with the points raised by the reviewers in the 'Response to Reviewers' box. If you do not agree with any of their criticisms or suggestions please explain clearly why this is so.

Reviewer 1

Advance summary and potential significance to field

This manuscript elegantly describes mechanisms of hair cell turnover and regeneration in the zebrafish inner ear. Although the zebrafish lateral line has been commonly leveraged as a model

© 2024. Published by The Company of Biologists under the terms of the Creative Commons Attribution License (https://creativecommons.org/licenses/by/4.0/).

hair cell sensory system, the zebrafish inner ear has been understudied. Here, Beaulieu and colleagues show that the number of HCs in the zebrafish inner ear steadily increases across larval development, and that HC turnover is quite low in the inner ear as compared to the zebrafish lateral line. They then utilize a new tool to induce damage to inner ear hair cells and demonstrate that there are key differences in the regenerative program in the inner ear as compared to the lateral line. They find that injury in the inner ear is followed by an initial early phase of SC division to expand the pool of progenitor cells and that the number of HCs recovers over time through continuous transdifferentiation of SCs. The rate of transdifferentiation of SCs (compared to the total # of SCs) is not increased following injury, and newly divided progenitor cells are not more likely to transdifferentiate into HCs than older progenitors. Overall, this study executes thoughtful and well-controlled experiments to characterize the mechanisms of HC proliferation and regeneration in a powerful genetic model of the inner ear.

Comments for the author

Major Concerns/suggestions:
• The myo6b:TrpV1-mClover has not been previously published. The authors should validate this tool by showing that there are no baseline cytotoxic effects (HC/SC loss or fewer HCs/SCs) -in the absence of capsaicin.
In addition, it may also be helpful to also show that there is no obvious damage to SCs after capsaicin treatment – for example, show that SC number/morphology appear broadly normal following capsaicin treatment.
• Are both cabp1b+ and cabp1- hair cells ablated (or scn5lab) to the same extent in the myo6b:TrpV1-mClover/Capsaicin paradigm? If one population is more susceptible to damage this could skew the interpretation of cell identify during regeneration.
• Did the authors consider non-linear regression models to explain the rate of HC addition during normal development (Fig. 2)? To claim linear increases over time, it would be best to show that linear models produce a better fit than non-linear alternatives.
• Fig. 8 and Fig. 9 should show examples from more than one timepoint.
Fig. 8 can then be referred to more heavily for the “Hair cells regenerate primarily by transdifferentiation” section of the text.
• The claim that the progenitor response is “uncoupled” from transdifferentiation is a bit misleading? This statement requires a manipulation within regenerating organs (i.e. not just comparing steady-state organs to regenerating ones). For example, blocking progenitor pool expansion in a regenerating organ and observing that HC number does not fully recover because the rate of transdifferentation is constant and the size of the progenitor pool is not expanded. The authors show that a cell’s proliferation state during early stages of regeneration doesn’t predict later transdifferentiation, but this is not quite the same as showing that the two events are uncoupled. This critique may seem picky, but the “uncoupled” terminology is in the title of the paper is a bit misleading and needs better support.

Minor Concerns/suggestions:
• The authors use “progenitor pool” and “SCs” largely interchangeably in the text. It would be helpful to delineate these more clearly, in alignment with the idea from RNAseq data that SCs and progenitors might be two separable populations.
• Although not 100% necessary, it would be nice to have evidence that in Fig. 4 – to show that scn5lab- HCs become scn5lab+ over time.
• Line 83 specific vestibular sensory organs
• Line 88-90 This is a bit confusing. In mammals there are distinct physical that define extrastriolar and extrastriolar domains rather than a specific cell type (there are type 1 and II cells in each region). In addition innervation dimorphism defines these regions. But in zebrafish these regions are defined by specific hair cell types. Is this correct?
• Line 93 Cristae hair cells are also morphologically in zebrafish as well (tall and short) (Smith et al 2020).
• Line 205 Need a bit more context here. When where nascent HCs identified? When were the fish treated?
• Line 218 for the control groups, were double transgenic fish later identified as controls after the completion of the experiment (via PCR for mClover) or did the control population remain mixed?
• Line 298: after hair cell ABLATION

© 2024. Published by The Company of Biologists under the terms of the Creative Commons Attribution License (https://creativecommons.org/licenses/by/4.0/).

Figures and methods:

- Fig. 3C caption: (n = 20 control, 20 ablated) should be converted, not ablated
- "The increased ratio for old cells suggests phenotypic conversion from peripheral to central hair cell type [need a space here] over time."
- What is the concentration of capsaicin used in Fig. 5A-C? And how many hpa is the graph in D?
- Fig. S3 – the labels on the images should be consistent with the rest of the paper – dpa, not dpt.
- Please add the NA of your objective lenses.
- Please add "photoconvert" to your Fig. 8A schematics as you have for Fig. 9. It really helps distinguish the two experiments and very much helps make sense of Table 1 (for ex, why the "new HC" counts are so different between the Fig. 8 and Fig. 9 data).
- Why is there no data for the number of new HCs at 1dpa for Fig. 8 data in Table 1?
- Please add scale bars for 1 dpa and 2 dpa data in Fig. 6.

Reviewer 2

Advance summary and potential significance to field

Beaulieu and colleagues have submitted a manuscript that addresses a longstanding question in developmental biology concerning the mechanisms behind hair cell regeneration. Two mechanisms have been proposed to explain hair cell regeneration – mitosis or transdifferentiation – in various animal models, but the exact process of hair cell regeneration in vivo remains elusive. Using the zebrafish Beaulieu et al. evaluate hair cell growth over time and show that hair cells grow in the absence of continuous turnover. They use a well-established transgenic zebrafish which expresses a photoconvertible protein in hair cell nuclei to determine the rate of hair cell addition in the inner ear as the larvae grow over time and to evaluate hair cell turnover based on the presence or absence of photoconvertible protein in hair cell nuclei. Excitingly, they demonstrate that hair cell regeneration occurs through transdifferentiation in the larval inner ear via a wave of supporting cell or progenitor cell proliferation in response to hair cell death. They use an innovative genetically encoded method for hair cell ablation combined with transgenic lines to examine regenerated hair cells. By examining proliferation using a well-established approach, EdU incorporation, they demonstrate that transdifferentiation and not proliferation is the primary mode of hair cell regeneration. Finally, they detect an expansion of supporting cells or progenitor cells in response to hair cell ablation that do not contribute directly to new hair cells. This is very interesting work as it critically evaluates and tests genomic insights in a way that is convincing. I find the work carefully done and well supported with a couple exceptions that I believe can strengthen some of the observed results.

Comments for the author

Beaulieu and colleagues have submitted a manuscript that addresses a longstanding question in developmental biology concerning the mechanisms behind hair cell regeneration. Two mechanisms have been proposed to explain hair cell regeneration – mitosis or transdifferentiation – in various animal models, but the exact process of hair cell regeneration in vivo remains elusive. Using the zebrafish, Beaulieu et al. evaluate hair cell growth over time and show that hair cells grow in the absence of continuous turnover. They use a well-established transgenic zebrafish which expresses a photoconvertible protein in hair cell nuclei to determine the rate of hair cell addition in the inner ear as the larvae grow over time and to evaluate hair cell turnover based on the presence or absence of photoconvertible protein in hair cell nuclei. Excitingly, they demonstrate that hair cell regeneration occurs through transdifferentiation in the larval inner ear via a wave of supporting cell or progenitor cell proliferation in response to hair cell death. They use an innovative genetically encoded method for hair cell ablation combined with transgenic lines to examine regenerated hair cells. By examining proliferation using a well-established approach, EdU incorporation, they demonstrate that transdifferentiation and not proliferation is the primary mode of hair cell regeneration. Finally, they detect an expansion of supporting cells or progenitor cells in response to hair cell ablation that do not contribute directly to new hair cells. This is very interesting work as it critically evaluates and tests genomic insights in a way that is convincing. I

© 2024. Published by The Company of Biologists under the terms of the Creative Commons Attribution License (https://creativecommons.org/licenses/by/4.0/).

find the work carefully done and well supported with a couple exceptions that I believe can strengthen some of the observed results.

1) Comment related to Zebrafish inner ear sensory patches grow constantly during the larval stage: The authors very clearly show that hair cells are added during larval inner ear growth (Fig 2 and Fig 4D) and there is little hair cell turnover (Fig 3). In the discussion it is mentioned that during growth, supporting cells convert to hair cells using mechanisms uncoupled from cell division. The authors examine proliferation using a well-established technique, EdU labeling. However, it is unclear to me that hair cells are added during growth via transdifferentiation or from cell division/proliferation. Do the authors also observe an absence of EdU-labeling of hair cells or supporting cells over the course of inner ear growth?

2) Comments related to representation of Figure 8: The image in the Fig 8C Control EdU+ has maybe 12-13 cells that are EdU positive. The graph in figure 8B claims an average of 35 or so EdU positive supporting cells and the lowest number being 25 according to error bars. The image in Fig 8C doesn't represent the graphed data collected. A more representative image of the data collected should be shown.

3) Comments related to Figure 8: Excitingly, the authors show evidence that non-sensory cells proliferate in response to hair cell ablation (Fig 8B,C). The authors raise the intriguing idea in the discussion (281-282) that "supporting cells that divided in response to hair cell ablation are not more likely to differentiate into hair cells". I will add that it is unclear if there is a progenitor pool of a non-sensory supporting cell type that exists in the epithelium and these cell types are actively dividing to become HC (or become SC). This does require further experimental evaluation that may not fit within the scope of this manuscript. However, addressing the identities of the supporting cell population as well as the hair cell subtypes will strengthen the manuscript. Are supporting cells in Fig 8C indeed supporting cells (Sox2+ by immunostaining or zpld1a+ by HCR) or progenitors (dla+ by HCR)? If there is a progenitor pool expansion during regeneration, this will be supported by HCR or in situ to label this population in the inner ear using validated markers. In addition, Sox2+ supporting cells transdifferentiate to give rise to new hair cells (Millimaki, 2020). An additional test demonstrating whether the populations of EdU+ supporting cells is Sox2+ or not will support and strengthen the exciting conclusions that support cells transdifferentiate to regenerate hair cells in the inner ear.

4) Comments related to Figure 9: In the legend "unconverted Eos (cyan), and without photoconverted eos (magenta)." In Fig 9B, magenta text says conv. Eos and this might be a typo. The Conv. Eos in magenta is difficult to see in Fig 9B in comparison to Figure 7 A,C. It would be helpful to have a panel with magenta cells only like Figure 7 (just by splitting the channels).

<u>Reviewer 3</u>

Advance summary and potential significance to field

In this manuscript by Beaulieu, et al., the authors characterize the growth and regeneration of mechanosensory hair cells in the larval zebrafish inner ear. Using transgenic and histological approaches they assess the proliferation and differentiation of surrounding supporting cells at multiple timepoints during normal growth and following hair cell ablation. The authors observe an immediate proliferative response in supporting cells after ablation, followed by a gradual replacement and patterning of hair cells that occurs under dynamics similar to normal growth. From these data the authors conclude that the two responses are largely uncoupled, and that regeneration occurs through a transient increase in the number of progenitor cells available for differentiation. Overall, this is a very solid paper with only its potential impact in question. While the manuscript will likely be of wide interest, and the findings are novel with respect to the process of neuromast hair cell regeneration, it is primarily a descriptive characterization that provides limited mechanistic insight that could advance the field.

Comments for the author

© 2024. Published by The Company of Biologists under the terms of the Creative Commons Attribution License (https://creativecommons.org/licenses/by/4.0/).

This study was carefully designed and reported, with clearly presented data that support the authors' conclusions. The work will help establish the zebrafish larva as new model for inner ear hair cell regeneration and lays a solid foundation for comparison with other regenerative models. I have no significant concerns or recommendations for publication, other than to suggest that the inclusion of a model figure could help summarize the authors' interpretations of their data and extensive comparisons with other regenerative mechanisms outlined in the Discussion.

First revision

Author response to reviewers' comments

We thank the reviewers for their thoughtful critique of our work. We address their points individually below:

Reviewer 1 Comments for the author
Major Concerns/suggestions:
•The myo6b:TrpV1-mClover has not been previously published. The authors should validate this tool by showing that there are no baseline cytotoxic effects (HC/SC loss or fewer HCs/SCs) -in the absence of capsaicin. In addition, it may also be helpful to also show that there is no obvious damage to SCs after capsaicin treatment – for example, show that SC number/morphology appear broadly normal following capsaicin treatment.
We now include hair cell counts from WT and myo6b:TrpV1-mClover (het) fish at 8 dpf in Figure S4. We find there is no difference in hair cell number, suggesting no cytotoxic effect of the transgene. We also present hair cell and supporting cell counts from control and ablated fish at 8 dpf (Figure S4). We find that supporting cell number is broadly normal after damage.

•Are both cabp1b+ and cabp1- hair cells ablated (or scn5lab) to the same extent in the myo6b:TrpV1-mClover/Capsaicin paradigm? If one population is more susceptible to damage this could skew the interpretation of cell identify during regeneration.
We have not directly investigated the identity of surviving hair cells in ablated cristae, as ablation is nearly complete, with less than 10% remaining (Fig 5; Fig S4). However our use of the photoconvertible marker allows us to distinguish the few remaining hair cells and identify new regenerated hair cells in our analysis.

•Did the authors consider non-linear regression models to explain the rate of HC addition during normal development (Fig. 2)? To claim linear increases over time, it would be best to show that linear models produce a better fit than non-linear alternatives.
As the reviewer suggested, we tested non-linear alternative models for hair cell addition during development. A linear model was best in all tests for the lateral crista and utricle, but it is possible that a non-linear model would best fit the anterior crista. As such, we have removed the linear regression from the figure and text.

•Fig. 8 and Fig. 9 should show examples from more than one timepoint. Fig. 8 can then be referred to more heavily for the "Hair cells regenerate primarily by transdifferentiation" section of the text.
We now include images from additional timepoints in Figures S8 and S11.

•The claim that the progenitor response is "uncoupled" from transdifferentiation is a bit misleading? This statement requires a manipulation within regenerating organs (i.e. not just comparing steady-state organs to regenerating ones). For example, blocking progenitor pool expansion in a regenerating organ and observing that HC number does not fully recover because the rate of transdifferentation is constant and the size of the progenitor pool is not expanded. The authors show that a cell's proliferation state during early stages of regeneration doesn't predict later transdifferentiation, but this is not quite the same as showing that the two events are uncoupled. This critique may seem picky, but the "uncoupled" terminology is in the title of the paper is a bit misleading and needs better support.
We have narrowed our definition of coupled/uncoupled to describe the temporal differences in events. We believe that the two observed events – a transient wave of supporting cell proliferation

© 2024. Published by The Company of Biologists under the terms of the Creative Commons Attribution License (https://creativecommons.org/licenses/by/4.0/).

and supporting cell transdifferentiation into hair cells – are temporally uncoupled. First, the timing of the two events is different, with a transient wave of proliferation and a sustained process of transdifferentation. Second, although we see an increase in the overall number of EdU-labeled hair cells derived from supporting cell proliferation, the fraction of labeled hair cells is no different after damage than during growth. This suggests that supporting cells induced to divide after damage are no more likely to differentiate as hair cells. We agree that additional experiments are necessary and include this point in the discussion (line 342-343).

Minor Concerns/suggestions:
•The authors use “progenitor pool” and “SCs” largely interchangeably in the text. It would be helpful to delineate these more clearly, in alignment with the idea from RNAseq data that SCs and progenitors might be two separable populations.
To address this point, have substituted “supporting cells” for “progenitor pool” in cases where we do not clearly define differences, as “supporting cells” is the broader definition including progenitors.

•Although not 100% necessary, it would be nice to have evidence that in Fig. 4 – to show that scn5lab- HCs become scn5lab+ over time.
We now include representative images with scn5lab HCR in Figure S3. These images confirm that at 2 dpp many photoconverted cells are outside the central scn5lab+ zone, but by 14dpp almost all photoconverted HC nuclei are located within the scn5lab+ zone.

•Line 83 specific vestibular sensory organs
Corrected

•Line 88-90 This is a bit confusing. In mammals there are distinct physical that define extrastriolar and extrastriolar domains rather than a specific cell type (there are type 1 and II cells in each region). In addition, innervation dimorphism defines these regions. But in zebrafish these regions are defined by specific hair cell types. Is this correct?

We have re-written this section to better describe striolar and extrastiolar regions across species (lines 92-97).

•Line 93 Cristae hair cells are also morphologically in zebrafish as well (tall and short) (Smith et al 2020).
Corrected and reference added

•Line 205 Need a bit more context here. When where nascent HCs identified? When were the fish treated?
Reworded for clarification lines 207-209

•Line 218 for the control groups, were double transgenic fish later identified as controls after the completion of the experiment (via PCR for mClover) or did the control population remain mixed?
This sentence has been reworded to clarify the screening of ablated vs control fish (Lines 221-223). The extent of hair cell destruction in the TrpV-mClover+ fish was complete enough that there was not an intermediate phenotype (i.e. with only a few hair cells dying), so we did not feel it was necessary to confirm by PCR that larvae without dying hair cells lacked the TrpV-mClover transgene.

•Line 298: after hair cell ABLATION
Fixed

Figures and methods:
•Fig. 3C caption: (n = 20 control, 20 ablated) should be converted, not Ablated
Fixed – line 803, actually it should be the two timepoints for the turnover experiments: 8 dpf and 15 dpf

•“The increased ratio for old cells suggests phenotypic conversion from peripheral to central hair cell type [need a space here] over time.”
Fixed – line 816

© 2024. Published by The Company of Biologists under the terms of the Creative Commons Attribution License (https://creativecommons.org/licenses/by/4.0/).

•What is the concentration of capsaicin used in Fig. 5A-C? And how many hpa is the graph in D?
Fixed – lines 208, 215-218

•Fig. S3 – the labels on the images should be consistent with the rest of the paper – dpa, not dpt.
Labels have been changed to dpa (now Figure S5).

•Please add the NA of your objective lenses.
NA of objective lenses has been added – lines 485-489

•Please add "photoconvert" to your Fig. 8A schematics as you have for Fig. 9. It really helps distinguish the two experiments and very much helps make sense of Table 1 (for ex, why the "new HC" counts are so different between the Fig. 8 and Fig. 9 data).
Done

•Why is there no data for the number of new HCs at 1dpa for Fig. 8 data in Table 1?
For the 0-1 dpa EdU pulse, we used DAPI to label cell nuclei, as shown in Fig 8C. Imaging with the 488 laser will photoconvert Eos, so we could not be confident in identifying new vs preexisting hair cells while also imaging DAPI. We determined after this timepoint that DAPI was not necessary and that counting EdU+ support cells could be done based on nucleus shape and proximity to hair cells, so we were able to count new unconverted-only Eos+ hair cells for the 3-4 and 6-7 day EdU pulses.

•Please add scale bars for 1 dpa and 2 dpa data in Fig. 6.
Scale bars have been added all timepoints in Figure 6 and S5.

Reviewer 2 Comments for the author
1) Comment related to Zebrafish inner ear sensory patches grow constantly during the larval stage: The authors very clearly show that hair cells are added during larval inner ear growth (Fig 2 and Fig 4D) and there is little hair cell turnover (Fig 3). In the discussion it is mentioned that during growth, supporting cells convert to hair cells using mechanisms uncoupled from cell division. The authors examine proliferation using a well-established technique, EdU labeling. However, it is unclear to me that hair cells are added during growth via transdifferentiation or from cell division/proliferation. Do the authors also observe an absence of EdU-labeling of hair cells or supporting cells over the course of inner ear growth?
We see a similar pattern of hair cell addition during growth. We observe supporting cell proliferation under growth conditions (Fig 8, Fig S12), but substantial delay before EdU labeling is incorporated into hair cells (Fig 9).

2) Comments related to representation of Figure 8: The image in the Fig 8C Control EdU+ has maybe 12-13 cells that are EdU positive. The graph in figure 8B claims an average of 35 or so EdU positive supporting cells and the lowest number being 25 according to error bars. The image in Fig 8C doesn't represent the graphed data collected. A more representative image of the data collected should be shown.
When counting cells in the EdU experiments, EdU+ HC were so uncommon that we decided to combine counts for the anterior and lateral cristae to increase power. A sentence has been added to the results section to clarify this – lines 262-263

3) Comments related to Figure 8: Excitingly, the authors show evidence that non-sensory cells proliferate in response to hair cell ablation (Fig 8B,C). The authors raise the intriguing idea in the discussion (281-282) that "supporting cells that divided in response to hair cell ablation are not more likely to differentiate into hair cells". I will add that it is unclear if there is a progenitor pool of a non-sensory supporting cell type that exists in the epithelium and these cell types are actively dividing to become HC (or become SC). This does require further experimental evaluation that may not fit within the scope of this manuscript. However, addressing the identities of the supporting cell population as well as the hair cell subtypes will strengthen the manuscript. Are supporting cells in Fig 8C indeed supporting cells (Sox2+ by immunostaining or zpld1a+ by HCR) or progenitors (dla+ by HCR)? If there is a progenitor pool expansion during regeneration, this will be supported by HCR or in situ to label this population in the inner ear

© 2024. Published by The Company of Biologists under the terms of the Creative Commons Attribution License (https://creativecommons.org/licenses/by/4.0/).

using validated markers. In addition, Sox2+ supporting cells transdifferentiate to give rise to new hair cells (Millimaki, 2020). An additional test demonstrating whether the populations of EdU+ supporting cells is Sox2+ or not will support and strengthen the exciting conclusions that support cells transdifferentiate to regenerate hair cells in the inner ear.
The dividing cells that are located underneath and adjacent to the hair cells are indeed zpld1a+. Representative images of the 0-1dpa EdU pulse experiment with HCR for zpld1a have been added to Fig. S10.

4) Comments related to Figure 9: In the legend "unconverted Eos (cyan), and without photoconverted eos (magenta)." In Fig 9B, magenta text says conv. Eos and this might be a typo. The Conv. Eos in magenta is difficult to see in Fig 9B in comparison to Figure 7 A,C. It would be helpful to have a panel with magenta cells only like Figure 7 (just by splitting the channels).
The legend has been corrected and the converted Eos channel has been added to Figure 9.

Reviewer 3 Comments for the author
This study was carefully designed and reported, with clearly presented data that support the authors' conclusions. The work will help establish the zebrafish larva as new model for inner ear hair cell regeneration and lays a solid foundation for comparison with other regenerative models. I have no significant concerns or recommendations for publication, other than to suggest that the inclusion of a model figure could help summarize the authors' interpretations of their data and extensive comparisons with other regenerative mechanisms outlined in the Discussion.
A summary figure has been added in Figure S13

<u>Second decision letter</u>

MS ID#: DEVELOP/2024/202944

MS TITLE: Transdifferentiation is temporally uncoupled from progenitor pool expansion during hair cell regeneration in the zebrafish inner ear

AUTHORS: Marielle O. Beaulieu, Eric D. Thomas, and David W Raible
ARTICLE TYPE: Research Article

I am happy to tell you that your manuscript has been accepted for publication in Development, pending our standard publication integrity checks.

<u>Reviewer 1</u>

Advance summary and potential significance to field

This manuscript elegantly describes mechanisms of hair cell turnover and regeneration in the zebrafish inner ear. Although the zebrafish lateral line has been commonly leveraged as a model hair cell sensory system, the zebrafish inner ear has been understudied. Here, Beaulieu and colleagues show that the number of HCs in the zebrafish inner ear steadily increases across larval development, and that HC turnover is quite low in the inner ear as compared to the zebrafish lateral line. They then utilize a new tool to induce damage to inner ear hair cells and demonstrate that there are key differences in the regenerative program in the inner ear as compared to the lateral line. They find that injury in the inner ear is followed by an initial early phase of SC division to expand the pool of progenitor cells and that the number of HCs recovers over time through continuous transdifferentiation of SCs. The rate of transdifferentiation of SCs (compared to the total # of SCs) is not increased following injury, and newly divided progenitor cells are not more likely to transdifferentiate into HCs than older progenitors. Overall, this study executes thoughtful and well-controlled experiments to characterize the mechanisms of HC proliferation and regeneration in a powerful genetic model of the inner ear.

Comments for the author

© 2024. Published by The Company of Biologists under the terms of the Creative Commons Attribution License (https://creativecommons.org/licenses/by/4.0/).

The authors have addressed all my suggestions!

Reviewer 2

Advance summary and potential significance to field

Beaulieu and colleagues have submitted a manuscript that addresses a longstanding question in developmental biology concerning the mechanisms behind hair cell regeneration. Two mechanisms have been proposed to explain hair cell regeneration – mitosis or transdifferentiation – in various animal models, but the exact process of hair cell regeneration in vivo remains elusive. Using the zebrafish Beaulieu et al. evaluate hair cell growth over time and show that hair cells grow in the absence of continuous turnover. They use a well-established transgenic zebrafish which expresses a photoconvertible protein in hair cell nuclei to determine the rate of hair cell addition in the inner ear as the larvae grow over time and to evaluate hair cell turnover based on the presence or absence of photoconvertible protein in hair cell nuclei. Excitingly, they demonstrate that hair cell regeneration occurs through transdifferentiation in the larval inner ear via a wave of supporting cell or progenitor cell proliferation in response to hair cell death. They use an innovative genetically encoded method for hair cell ablation combined with transgenic lines to examine regenerated hair cells. By examining proliferation using a well-established approach, EdU incorporation, they demonstrate that transdifferentiation and not proliferation is the primary mode of hair cell regeneration. Finally, they detect an expansion of supporting cells or progenitor cells in response to hair cell ablation that do not contribute directly to new hair cells. This is very interesting work that moves the field forward as it critically evaluates and tests genomic insights in a way that is convincing.

Comments for the author

The authors addressed the four comments/concerns raised by clarifying results in text. They also added essential data to support claims (Fig S10). There are no additional comments for this version of the manuscript.
The manuscript is suitable for publication and contributes to the field.

© 2024. Published by The Company of Biologists under the terms of the Creative Commons Attribution License (https://creativecommons.org/licenses/by/4.0/).
